# Risk factors for recurrences and visual impairment in patients with ocular toxoplasmosis: A systematic review and meta-analysis

**DOI:** 10.1371/journal.pone.0283845

**Published:** 2023-04-03

**Authors:** Carlos Cifuentes-González, William Rojas-Carabali, Álvaro Olate Pérez, Érika Carvalho, Felipe Valenzuela, Lucía Miguel-Escuder, María Soledad Ormaechea, Milagros Heredia, Pablo Baquero-Ospina, Alfredo Adan, Andre Curi, Ariel Schlaen, Cristhian Alejandro Urzua, Cristóbal Couto, Lourdes Arellanes, Alejandra de-la-Torre

**Affiliations:** 1 Neuroscience (NEUROS) Research Group, Neurovitae Center for Neuroscience, Institute of Translational Medicine (IMT), Escuela de Medicina y Ciencias de la Salud, Universidad del Rosario, Bogotá, Colombia; 2 Laboratory of Ocular and Systemic Autoimmune Diseases, Faculty of Medicine, University of Chile, Santiago, Chile; 3 Clinical Research Laboratory of Infectious Diseases in Ophthalmogy, National Institute of Infectious Disease, INI—Oswaldo Cruz Foundation, Rio de Janeiro, Brazil; 4 Hospital Clinic of Barcelona, Clinic Institute of Ophthalmology, University of Barcelona, Barcelona, Spain; 5 Department of Ophthalmology, Hospital de Clinicas Jose de San Martin, Universidad de Buenos Aires, Buenos Aires City, Argentina; 6 Department of Ophthalmology, Hospital Universitario Austral, Buenos Aires, Argentina; 7 Inflammatory Eye Disease Clinic, Dr. Luis Sanchez Bulnes" Hospital, Asociación para Evitar la Ceguera en México (APEC), Mexico City, CDMX, Mexico; 8 Red de Investigación en Inmunología Ocular de Latinoamérica (RIOLAT); 9 Faculty of Medicine, Clinica Alemana-Universidad del Desarrollo, Santiago, Chile; Taipei Veterans General Hospital | National Yang Ming Chiao Tung University, TAIWAN

## Abstract

**Background:**

Ocular toxoplasmosis (OT) is caused by the parasite *Toxoplasma gondii*. OT is the leading cause of posterior uveitis globally; it is a recurrent disease that may result in visual impairment and blindness. This systematic review and meta-analysis aim to summarize and evaluate the risk factors for recurrences, visual impairment, and blindness described in the literature worldwide.

**Methods and findings:**

We performed a systematic literature search in PubMed, Embase, VHL, Cochrane Library, Scopus, and DANS EASY Archive. All studies reporting patients with clinically and serologically confirmed OT presenting any clinical or paraclinical factor influencing recurrences, visual impairment, and blindness were included. Studies presenting secondary data, case reports, and case series were excluded. An initial selection was made by title and abstract, and then the studies were reviewed by full text where the eligible studies were selected. Then, the risk of bias was assessed through validated tools. Data were extracted using a validated extraction format. Qualitative synthesis and quantitative analysis were done. This study was registered on PROSPERO (CRD42022327836).

**Results:**

Seventy two studies met the inclusion criteria. Fifty-three were summarized in the qualitative synthesis in three sections: clinical and environmental factors, parasite and host factors, and treatment-related factors. Of the 72 articles, 39 were included in the meta-analysis, of which 14 were conducted in South America, 13 in Europe, four in Asia, three multinational, two in North America and Central America, respectively, and only one in Africa. A total of 4,200 patients with OT were analyzed, mean age ranged from 7.3 to 65.1 year of age, with similar distribution by sex. The frequency of recurrences in patients with OT was 49% (95% CI 40%–58%), being more frequent in the South American population than in Europeans. Additionally, visual impairment was presented in 35% (95% CI 25%–48%) and blindness in 20% (95% CI 13%–30%) of eyes, with a similar predominance in South Americans than in Europeans. On the other hand, having lesions near the macula or adjacent to the optic nerve had an OR of 4.83 (95% CI; 2.72–8.59) for blindness, similar to having more than one recurrence that had an OR of 3.18 (95% CI; 1.59–6.38). Finally, the prophylactic therapy with Trimethoprim/Sulfamethoxazole versus the placebo showed a protective factor of 83% during the first year and 87% in the second year after treatment.

**Conclusion:**

Our Systematic Review showed that clinical factors such as being older than 40 years, patients with de novo OT lesions or with less than one year after the first episode, macular area involvement, lesions greater than 1 disc diameter, congenital toxoplasmosis, and bilateral compromise had more risk of recurrences. Also, environmental and parasite factors such as precipitations, geographical region where the infection is acquired, and more virulent strains confer greater risk of recurrences. Therefore, patients with the above mentioned clinical, environmental, and parasite factors could benefit from using prophylactic therapy.

## Introduction

Ocular Toxoplasmosis (OT) is caused by the parasite of the apicomplexan family known as *Toxoplasma gondii* (*Tg*), considered a neglected tropical disease [1]. Moreover, OT is the leading cause of posterior uveitis worldwide; its prevalence and clinical features varies according to region due to the different host and parasite factors [2–4].

Recurrences may occur in 40% to 79% of cases, generating visual impairment and potentially blindness [5–7]. The best treatment for OT is controversial and, there is no consensus on which drugs must be used for prophylactic treatment of recurrences [8–11]. Although, some risk factors for recurrences, such as the number of lesions (patients with only one lesion have a 60% greater risk than those with more than 2 lesions) [12], presence of polymorphisms (the presence of T/A heterozygosis in the IFN-γ gene at position AT+874 had a 49% higher risk of recurrences than those with A/A homozygosis) [12], and age (higher risk of recurrences was associated with older patients that present a de novo active lesion) [12–14] have been identified, it is still unclear which patients should receive prophylaxis.

Additionally, some data comes from studies with low-quality evidence, generating weak recommendations regarding risk factors for recurrence and visual outcomes, treatment and prophylactic treatment in OT. Thus, this topic deserves an update and synthesis of the available literature to provide high-quality evidence that leads to strong recommendations. Therefore, this systematic review and meta-analysis aimed to evaluate the factors that increase the risk of recurrence, visual impairment, and blindness in patients with OT.

## Methods

This systematic review and meta-analysis was conducted according to the Preferred Reporting Items for Systematic Reviews and Meta-Analyses (PRISMA) guidelines (**S1 Checklist)** [15]. It was registered in the “International prospective register of systematic reviews” (PROSPERO ID: CRD42022327836). Due to the characteristics of our study, it does not need to have ethical committee approval.

### Search strategy

We conducted a systematic literature search in the following databases: PubMed, Embase, VHL (Virtual Health Library), Cochrane Library (Ovid), Scopus, and DANS EASY Archive. We used “MeSH,” “Emtree, and “DeCS” terms accordingly. The search strategy can be found in **S1 Table**. We identified and deleted duplicated articles with the assistance of Zotero and Excel filters.

### Selection criteria

We reviewed papers that evaluated patients with a diagnosis of OT confirmed by clinical and/or serological criteria exposed to any clinical or paraclinical factor influencing our outcomes of interest, including recurrences or visual impairment.

Studies eligible for inclusion in this systematic review included clinical trials (randomized/non-randomized) and observational studies (case-control, cohort, cross-sectional studies, and population-based studies). We excluded secondary studies (systematic, narrative, and scoping reviews), case reports, case series, and articles from preclinical studies (not providing clinical outcome data) and abstracts.

### Definitions

Recurrence of OT was defined as the presence of an active retinochoroidal lesion associated with scarring in at least one eye (congenital and acquired) [7]. Additionally, the World Health Organization definitions for visual impairment and blindness were used [16].

### Selection process

**Fig 1:**
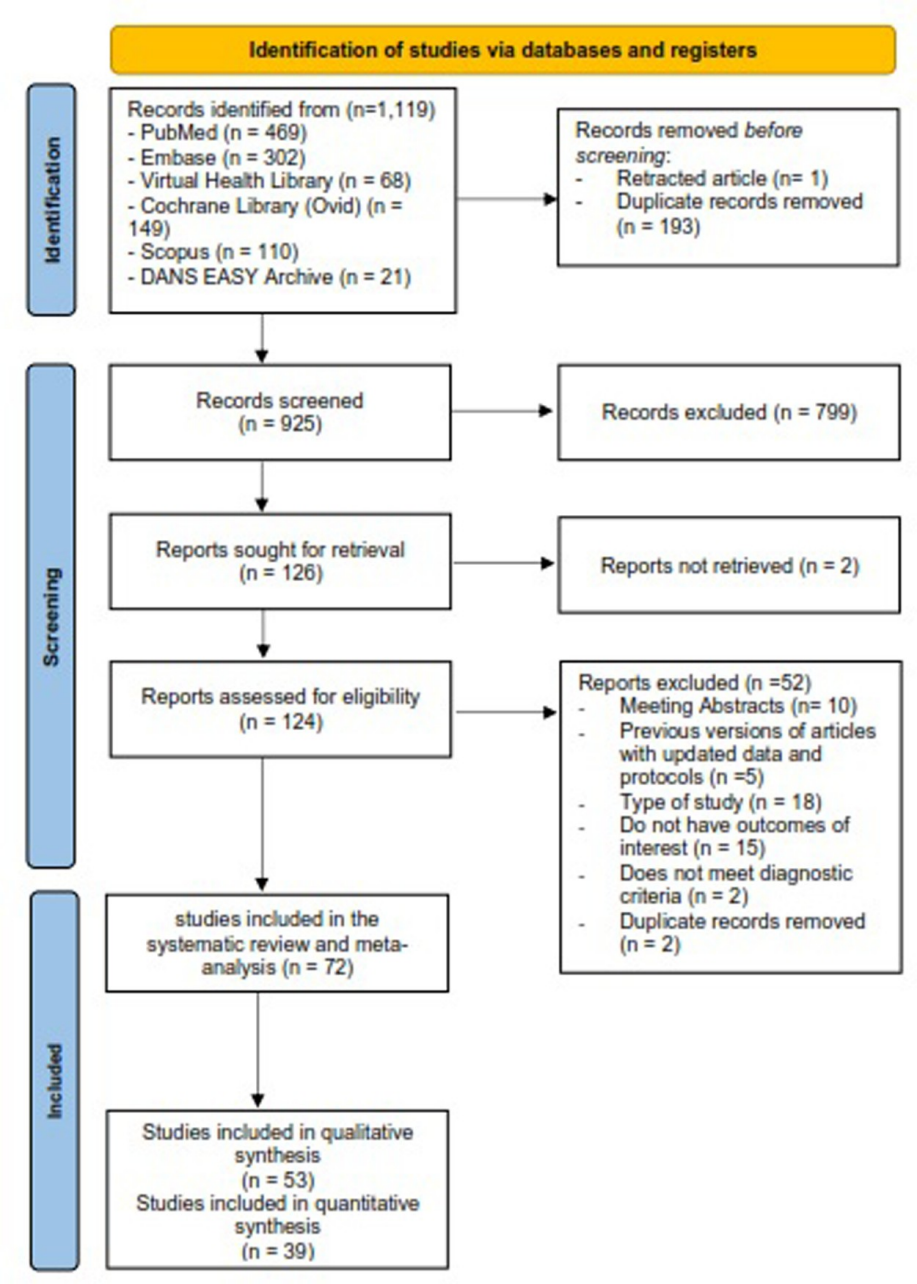
PRISMA flow diagram for selected studies included in the systematic review and meta-analysis.

Nine authors (AO, CC, EC, FV, LM, MS, MH, PB, WR) formed five pairs of review authors how independently examined the titles and abstracts identified by the electronic searches. Each author screened titles and abstracts independently to exclude those that were unrelated to our question based on the selection criteria; then, the independent decision was compared with the pair, and any disagreements were discussed. If disagreements persisted, a panel of seven thematic experts decided if the article should be included or excluded. The level of agreement in each couple was: C1 = 81.1% (LM+PB), C2 = 82.2% (FV+EC), C3 = 72.4% (MS+CC), C4 = 79.4% (MH+WR), and C5 = 96.2% (AO+WR). The overall agreement was 82.4%. The step-by-step can be found in **Fig 1.**

### Data extraction

Data were extracted by nine independent investigators (AO, CC, EC, FV, LM, MS, MH, PB, WR) using a standardized and validated Excel form. The following characteristics were extracted from each eligible study: article code, author, year, digital object identifier system, design of the study, intervention, country, studied population, age, race, sex, immunological status, *Tg* serotype/genotype, ocular inflammation characteristics, number of reactivations, factors related to the reactivations and magnitude of the risk, frequency of visual impairment and blindness, factors related to the visual impairment and blindness and magnitude of the risk, treatment, duration of treatment, type of prophylactic treatment, duration of prophylactic treatment, and sociodemographic factors. For all included studies, mean ± standard deviation (SD) or median and interquartile range (IQR) were used for data extraction.

### Risk of bias assessment and quality assessment

To assess the risk of bias for randomized clinical trials (RCTs), the version 2 of the Cochrane risk-of-bias tool for randomized trials (RoB 2) was used [17]. This tool includes five specific bias domains: randomization; deviation from intended intervention; missing data; outcome measurement; and selection of reported results. To evaluate the risk of bias in Non-Randomized studies of interventions, the ROBINS-I tool was used [18], which considered seven domains: confounding; selection of participants; classiﬁcation of intervention; deviation from interventions; missing outcome data; measurement of outcomes; selection of reported result. The figures were done using the Risk-of-bias VISualization (robvis) [19].

Moreover, quality of cross-sectional studies was assessed by the Agency for Healthcare Research and Quality (AHRQ) tool [20]; the score was categorized as high risk of bias (0 to 4 score), moderate risk of bias (5 to 7 score), and low risk of bias (8 to 11 score) [21]. Finally, to evaluate the risk of bias in longitudinal studies, cohorts, and case-control, the checklist provided by the Clinical Advances Through Research and Knowledge Translation (CLARITY) group of McMaster University was used [22]. Detailed information can be found in **S2 Table.**

### Data synthesis and statistical analysis

For prevalence, a meta-analysis of forest plots was conducted using the R Package (dmetar version 0.0.9000) [23, 24], and for related factors, forest plots Review Manager (RevMan 5.4; The Nordic Cochrane Centre, The Cochrane Collection, Copenhagen, Denmark) were used. A random effects model was used for all analyses, considering the significant heterogeneity of data. Only variables that were reported by at least two included studies underwent meta-analysis.

Since our primary outcome was to evaluate the factors influencing the recurrences rate, visual impairment, and blindness, we initially looked for the frequency of recurrences, visual impairment, and blindness subdivided by continents. The precaution of not including randomized and non-randomized clinical trials was always considered since they do not represent the reality of the world situation as they are controlled studies. Moreover, for the frequency analyses of visual impairment and blindness, we analyzed the final visual acuities reported in longitudinal and cross-sectional studies, and data was managed in eye categories because most studies report the number of eyes instead of patients. Studies that did not provide this data were excluded from the meta-analysis and included in the systematic review.

The influence of the following characteristics in the primary outcomes was evaluated: laterality of the disease, retinal location, sex, number of lesions, complications, and type of treatment. This analysis was done considering the number of patients, not the number of eyes. In order to assess the heterogenicity, we used the I^2^ statistics test with 30% to 60% as representing moderate heterogeneity and 50% to 90% representing substantial heterogeneity. Publication bias was evaluated using funnel plots if there were more than 10 studies. Signiﬁcance was set at the level of P-value less than 0.05.

Finally, for the qualitative synthesis, we created tables summarizing the most important factors related to recurrences and visual outcomes (visual impairment or blindness). They were subdivided according to the following categories: clinical and environmental factors, parasite and host factors, and therapeutic factors. All articles that provided relevant data for qualitative analysis were summarized, regardless they were included or not in the meta-analysis.

## Results

### Study selection

We found 1,119 articles, of which 193 duplicates were eliminated, and one article was a retraction of a manuscript. Subsequently, 799 articles were excluded in the initial screening by title and abstract, and 126 articles were included for full-text review. However, two articles could not be obtained, a Polish and a French article published more than 20 years ago. Therefore, 124 articles were evaluated, of which 72 were eligible, 53 for the systematic review since their outcomes had information regarding visual acuity and recurrences, and 39 for meta-analysis since they had similar measures that allowed us to combine the individual results. The articles without information regarding visual acuity or recurrences were not included in the systematic review. More detailed information can be found in **Fig 1**.

### Study characteristics

**Fig 2:**
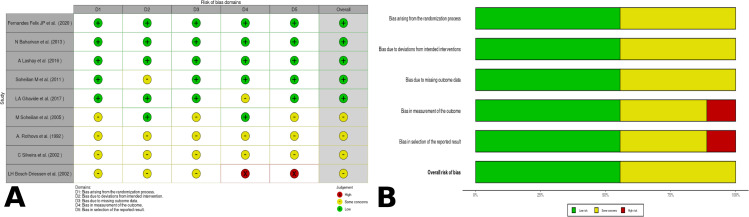
Risk of bias summary across randomized controlled trials using version 2 of the Cochrane risk-of-bias tool for randomized trials (RoB 2) [19]. (A). RoB2 risk of bias summary of randomized control trials traffic light; (B). Risk of bias graph across randomized controlled trials.

**Fig 3:**
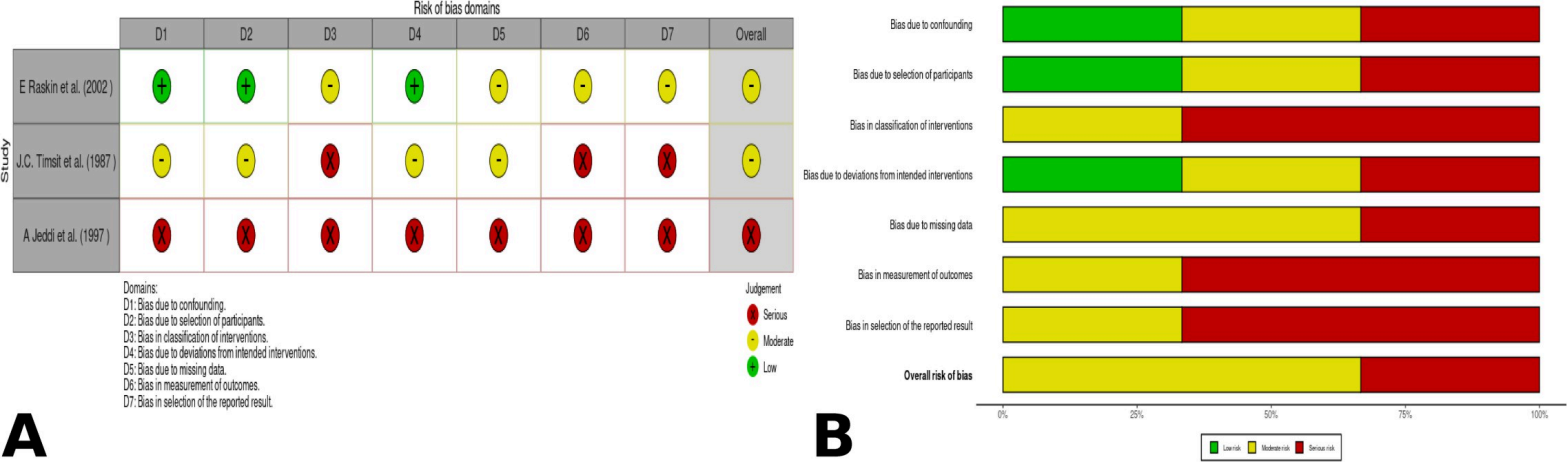
Summary across the Risk of Bias in Non-Randomized Studies of Interventions (ROBINS-I) tool [19]. (A). ROBINS-I risk of bias summary of non-randomized control trials traffic light; (B). ROBINS-I risk bias graph across non-randomized controlled trials.

Of the 72 analyzed studies, 9 were randomized clinical trials, which presented a low risk of bias in the outcomes of interest (**See Fig 2A and 2B**). On the other hand, three articles were non-randomized clinical trials that presented moderate to serious risks of bias for the outcomes (**See Fig 3A and 3B**). As for the cohort studies, 21 were analyzed and scored low risk of bias (an average of 6.90 out of 8 questions). Eight case-controls were identified and scored low risk of bias (an average of 4.87 out of 5 questions). Regarding the longitudinal studies, four were identified and scored low risk of bias (an average of 1.75 out of 3 questions). Finally, 27 cross-sectional articles were identified and scored moderate risk of bias (an average of 5.5 out of 11 questions) [5–7, 9, 11, 12, 14, 25–89].

In the quantitative analysis, 39 studies were used, 17 cross-sectional studies, 12 cohort studies, eight clinical trials (Two were non-randomized clinical trials), and two case-control studies. Of these, 39 were included in the meta-analysis, of which 14 were conducted in South America, 13 in Europe, four in Asia, three multinational, two in North America, two Central America, and only one in Africa. In total, 4,200 patients with OT were analyzed, mean age ranged from 7.3 to 65.1 year of age [Range 1 week to 87 years] (**S3 Table**) [7, 9, 12, 25–30, 32, 33, 35, 37–43, 48, 51–53, 55, 56, 62–64, 67–70, 72, 73, 78–80, 83, 85]. The remaining 53 articles were included in the qualitative summary, divided into clinical and environmental factors (**Table 1**), parasite factors (**Table 2**), and treatment-related factors (**Table 3**).

**Table 1:** Clinical and environmental factors.

Title	Authors (Year)	Continent	Type of Study	Factors related to recurrences in OT	Fractor that influence visual acuity outcome in OT
Clinical manifestations and visual outcomes associated with ocular toxoplasmosis in a Brazilian population	S Arruda et al. (2021) [27]	South America	Cohort	• Elderly patients (≥ 65 years old) had a higher risk of recurrence (OR: 1.02 (1.00–1.05) (*P* = 0.0493) • Active disease at presentation had a higher risk of recurrence (OR: 4.74 (1.95–12.91) (*P* = 0.0011)	Related factors to blindness on active OT at presentation • Atypical OT (OR: 4.99 (1.14–22.85) (*P* = 0.033) • Presence of any complication (OR:10.26 (3.82–30.67) (*P <* .0001) • Macular localization of the lesion (OR: 9.95 (2.45–47.15) *P* = 0.0019) Related factors to blindness on inactive OT at presentation • Lesion > 1DD (OR: 6.30 (2.28–22.46) *P* = 0.0013) • Macular localization of the lesion (OR: 9.95 (2.45–47.15) *P* < .0001)
Clinical and Multimodal Imaging Findings and Risk Factors for Ocular Involvement in a Presumed Waterborne Toxoplasmosis Outbreak, Brazil	C Brandão-de-Resende et al. (2014) [28]	South America	Cohort	Laterality: • Bilateral retinochoroidal involvement at baseline was statistically significantly associated with recurrences by log-rank test *(P* = 0.01) • All patients with bilateral involvement (n = 4) had recurrent lesions during the follow-up period compared with only 13% (1/8) of patients with monocular participation (RR = 8.0, 95% CI 1.3–50.0) (*P* = 0.01). Rate of recurrence: • 8.5% per person-month among patients with bilateral involvement and 0.4% per person-month among the patients with monocular participation (log-rank *P* = 0.01)	-
Ocular Involvement Following Postnatally Acquired Toxoplasma gondii Infection in Southern Brazil: A 28-Year Experience	TEF Arantes et al. (2015) [30]	South America	Cohort	Rate of recurrences The incidence of recurrences among patients with necrotizing retinochoroiditis at baseline or during follow-up was 10.5/100 per year	-
Influence of drug therapy on the risk of recurrence of ocular toxoplasmosis	M Reich et al. (2015) [31]	Europe	Cohort	Rate of recurrences • Median recurrence-free survival time of all 255 active lesions was 2.6 years (95% CI 2.1–3.0 years). • Median recurrence-free survival time in the subgroup of active lesions treated by *T*. *gondii*-specific antibiotics was 3.0 years (95% CI 2.2–3.9 years). • Median recurrence-free survival time in the subgroup treated with systemic corticosteroid monotherapy was 0.9 years (95% CI 0.5–1.3 years). • Median recurrence-free survival time in the subgroup treated without treatment was 3.0 years (95% CI 2.1 to 3.9 years) • Median recurrence-free survival time in the subgroup treated with non-specific antibiotic against *T*. *gondi* was 2.7 years (95% CI 1.2 to 4.3 years). • Risk of recurrence is significantly higher when treated with systemic corticosteroid monotherapy compared with *T*. *gondii*-specific antibiotic treatment (*P* <0.001) or compared with no therapy (*P* = 0.006). • No significant difference in median recurrence-free survival time between *T gondii*-specific antibiotic treatment versus no therapy was detected (*P* = 0.679).	-
[Epidemiology and clinical pattern of ocular Toxoplasmosis in Kinshasa]	NN Lusambo et al. (2019) [32]	Africa	Cross-sectional	-	• Visual impairment was associated with macular lesions (*P*< 0.001, OR = 3 (1,1,4–6) and age over 45 years (*P* = 0.002, OR = 11 (2.2–53.6). • Blindness was more frequent among patients with primary chorioretinal lesions (53.3%) compared to those with recurrence chorioretinal lesions (35.7%) and primary toxoplasmosis (33.3%) (*P* = 0.55).
Do Pregnancy, Postpartum Period, and Lactation Predispose Recurrent Toxoplasmic Retinochoroiditis?	J Brydak-Godowska et al. (2015) [33]	Europe	Cross-sectional	• Reactivation of OT during pregnancy has a RR of 7.40 IC 95% (4.46, 12.06) (*P* <0.0001), compared to the non-pregnant/non-lactating women.	-
Toxoplasmic retinochoroiditis: The influence of age, number of retinochoroidal lesions, and genetic polymorphism for IFN-γ +874 T/A as risk factors for recurrence in a survival analysis	ALQC Aleixo et al. (2019) [12]	South America	Cohort	• The risk of recurrence increased by 2% for each increase of 1 year of age (HR = 1.02), controlling other variables. • The risk of recurrence increased by 60% when the patients had one lesion (HR = 1.60, 95% CI = 1.07–2.40) compared to those with more than two lesions. The risk of recurrence increase 49% when there is AT heterozygosis for IFN-γ gene polymorphism at position +874 (HR = 1.49, 95% CI = 1.04–2.14), in contrast to patients that have AA homozygosis in the IFN-γ gene.	-
Clinical, Socio-economic, and Environmental Factors Related with Recurrences in Ocular Toxoplasmosis in Quindío, Colombia	S Velasco-Velásquez et al. (2020) [34]	South America	Longitudinal Studies	• Patients who reported intake of boiled water had a lower recurrence index (*P* = .042).	-
Toxoplasmic Retinochoroiditis: Clinical Characteristics and Visual Outcome in a Prospective Study	ALQC Aleixo et al. (2016) [35]	South America	Cross-sectional	-	Severe visual impairment was related to: • Posterior segment complications (*P* = 0.0035) • Localization of the lesion adjacent to the optic nerve and macula: (*P* = 0.0011) • Recurrences during follow (*P* = 0.0032)
Clinical pattern of ocular toxoplasmosis treated in a referral centre in Serbia	D Kovačević-Pavićević et al. (2012) [37]	Europe	Cross sectional	-	• At admission, patients with moderate visual impairment and poor treatment outcomes depended on the lesion localization (*P* <0.0001). • At admission, patients with severe visual impairment and poor treatment outcomes depended on the lesion localization (*P* = 0.006).
Frequency and factors associated with recurrences of ocular toxoplasmosis in a referral centre in Colombia	A de-la-Torre et al. (2009) [42]	South America	Cross-sectional	• The mean number of recurrences was two recurrences each 11 years. • The most important factors associated with recurrence were previous steroid therapy without antibiotics and previous subconjunctival steroid injection (*P* = 0.04 both). • A significant higher index of recurrences was found only in non-congenitally infected inactive cases (*P* = 0.006)	-
Analysis of Recurrence Patterns Associated with Toxoplasmic Retinochoroiditis	GN Holland et al. (2008) [44]	Europe	Cohort	• The recurrence rate was 0.2 episodes/ year or 1 episode/five years. • After a decade from the first episode of OT, the risk of recurrence risk decreases by 72% (aRR, 0.28; 95% CI, 0.22–0.36) (*P* < 0.001). • Also, disease duration remained strong when the multivariate analysis was repeated with all patients censored at 20 years of follow-up (aRR, 0.12 per 10-year increase in time; 95% CI, 0.09–0.16) (*P* = 0.001). • Patients over 40 years of age were at higher risk of recurrence than younger patients (RR 1.74; 95% CI, 1.06–2.86) (*P* = 0.03)	
Ocular Toxoplasmosis Clinical Features and Prognosis of 154 Patients	LEH Bosch-Driessen et al. (2002) [7]	Europe	Cross-sectional	• Recurrences occurred predominantly in previously affected eyes (with old scars) in contrast to the sporadic cases of recurrence in the healthy contralateral eye (*P* 0.0001).	• Legal blindness was more common in patients who had ever been treated with corticosteroids without antiparasitic drugs compared to those who received other or no treatments (*P* = 0.0004) • Legal blindness was more common in patients with a central location of the chorioretinal lesions compared to peripheral lesions (*P* < 0.0001) • Legal blindness was more common in extensive retinal lesions (>3DD) compared to lesions less than 3DD (*P* < 0.0001) • Poor visual outcome was more common in congenital toxoplasmosis than in patients with the acute phase of systemic infection (*P* <0.001) and also in patients with an unknown moment of acquisition of the infection (*P =* 0.03).
Risk of toxoplasmic retinitis reactivation following intraocular procedures without the use of prophylactic therapy	GC Heringer et al. (2014) [46]	South America	Cross-sectional	• Reactivation after the surgical procedure did not result in a significant reactivation rate (0.0008 in 17 months) of toxoplasmic retinochoroiditis.	-
Reactivations of Ocular Toxoplasmosis after Cataract Extraction	LH Bosch-Driessen et al. (2001) [47]	Europe	Case Controls	• Reactivation following cataract extraction had a higher incidence of recurrences than age -and sex-matched controls (*P* <0.001).	-
Recurrence characteristics in European patients with ocular toxoplasmosis	J G Garweg et al. (2008) [50]	Europe	Cross-sectional	• The interval between successive episodes remained stable between 1.0 and 1.7 years for the first three recurrences.	
Visual function in human ocular toxoplasmosis	J Scherrer et al. (2006) [51]	Europe	Cross-sectional	-	• Perimetry is a better option to identify the visual damage induced by OT, where moderate or severe visual field damage was encountered in 65.2% of eyes, and moderate or severe reduction of visual acuity in only 27.5% of eyes (*P* <0.001).
Posterior segment fndings by spectral‐domain optical coherence tomography and clinical associations in active toxoplasmic retinochoroiditis	GF Oliver et al. (2022) [52]	South America	Longitudinal Study	-	• Blindness was related to retinal destruction with hyporeflective spaces or signal voids, which was unusual in eyes achieving visual acuities of 20/40 or better, and present in half of the eyes retaining visual acuities of 20/200 or worse (*P* < 0.01).
Is reactivation of toxoplasmic retinochoroiditis associated to increased annual rainfall?	M Rudzinski et al. (2013) [54]	South America	Cohort	• For every mm of precipitation, there was a 2% increase in episodes (OR 1.002 CI 1.000–1.003).	-
Longitudinal Study of New Eye Lesions in Treated Congenital Toxoplasmosis	L Phan et al. (2007) [57]	North America	Cohort	-	• In congenital toxoplasmosis, there was not a statistically significant association between visual impairment at birth and development of a new chorioretinal lesion (*P* = 0.21)
Clinical Pattern of Toxoplasmic Retinochoroiditis in a Spanish Referral Center	A Rey et al. (2012) [64]	Multinational	Cross-sectional	-	• There were no statistically significant differences between the Spain vs. South America origin of patients for ocular visual prognosis. • Age ≥50 years at onset had an OR 3.27 (CI 1.22–8.8) (*P* <0.019) to have a final VA 20/200 • Initial VA ≤20/200 had an OR 63.47 (13.61–295.94) (*P* <0.001)to have a final VA 20/200 • The macular lesion had an OR 69.6 (8.31–583.1) (*P* <0.001) to have a final VA 20/200
Prevalence, clinical characteristics, and causes of vision loss in patients with ocular toxoplasmosis	NJS London et al. (2011) [65]	North America	Cross-sectional	-	• The main contributors to the visual loss were intraocular inflammation 74.8% and macular lesions 24.3%.
Risk of Visual Impairment in Children with Congenital Toxoplasmic Retinochoroiditis	HK Tan et al. (2007) [66]	Europe	Cohort	-	• Children with OT retinochoroiditis had a RR of 5.07 (95% CI, 2.62–9.81) (*P* < .0005) to have a visual impairment • In children with OT, the posterior pole lesion had a RR of 3.06 (95% CI, 1.16–9.28) (*P* = 0.031) to have a visual impairment.
Toxoplasmic retinochoroiditis and the evolution of visual results in immunocompetent patients	BT Naranjo Valladares et al. (2021) [70]	Central America	Cross-sectional	-	• When comparing each patient with himself at different follow-up momnets, there are differences in terms of BCVA, with a tendency to improve over time after treatment (*P* = 0.001). • It was identified that variables such as the location of the lesions in zone I, larger lesions >1 DD, and a high degree of inflammation were related to worse final BCVA.
Time patterns of recurrences and factors predisposing for a higher risk of recurrence of ocular toxoplasmosis	M Reich et al. (2015) [14] (10.1097/IAE.0000000000000361)	Europe	Cross-sectional	• The mean number of recurrences per year was 0.29 (SD 0.24). • The Median recurrence-free survival time was 2.52 years (95% Cl, 2.03–3.02 years). • Risk of recurrence was highest in the first year after the most recent episode (26%), implying a decrease with increasing recurrence-free intervals. • The recurrence risk decreased with the disease’s duration (*P* <0.001). • Treatment of the first active lesion influenced the risk of recurrence (*P* = 0.048). • Patient age at the time of the first active lesion (*P* = 0.021) and the most recent episode (*P* = 0.002) influenced the risk of recurrence	-
Recurrent Ocular Disease in Postnatally Acquired Toxoplasmosis	EH Bosch-Driessen et al. (1999) [76]	North America	Cross-sectional	• The number of patients with recurrences increased with the follow-up time: four (29%) of 14 during the first year of follow-up, eight (57%) of 14 during the second year, and eight of nine during the third follow-up year.	-
Active ocular Toxoplasmosis in Turkish patients: a report on 109 cases	I Tugal-Tutkun et al. (2005) [78]	Europe	Cohort	• Kaplan–Meier’s survival analysis estimated the risk of the first recurrence to be 7% at six months, 19% at 12 months, 35% at 24 months, 53% at 36 months, and 74% at 42 months.	-
Ocular Toxoplasmosis After the Fifth Decade	P Labalette et al. (2002) [79]	Europe	Cohort	-	• In patients older >50 years, lesion of >3DD determined a poor visual prognosis (*P* = 0.015)
Long-Term Ocular Prognosis in 327 Children With Congenital Toxoplasmosis	M Wallon et al. (2004) [85]	Europe	Cohort	• In congenital OT, the presence of 1 lesion in the first eye was predictive of the involvement of the partner eye (*P* = 0.0001).	-
Retinal Detachment in Ocular Toxoplasmosis	EH Bosch-Driessen et al. (1999) [6]	Europe	Cross-sectional	-	• In 5 of 9 patients with retinal detachment, a final VA20/200 was identified.
Ocular Toxoplasmosis: clinical-epidemiological aspects in pediatric age	BT Naranjo Valladares et al. (2020) [69]	South America	Cross sectional	-	• Lesion size >1DD and the localization of the lesson in zone I were associated with BCVA < or = 0.3 LogMar (*P* = 0.011 and *P* = 0.001, respectively).

BCVA: Best corrected visual acuity; CI: Confidence intervals; DD: Disc of diameter; VA: Visual acuity; OT: Ocular Toxoplasmosis; RR: Relative Risk; OR: Odds Ratio; HR: Hazard ratio

**Table 2:** Host and parasite factors related to recurrences and visual acuity.

Title	Authors (Year)	Continent	Type of Study	Factors related to recurrences in OT	Factor that influence visual acuity outcome in OT
Toxoplasmic retinochoroiditis: The influence of age, number of retinochoroidal lesions, and genetic polymorphism for IFN-γ +874 T/A as risk factors for recurrence in a survival analysis	ALQC Aleixo et al. (2019) [12]	South America	Cohort	• The risk of recurrence increases 49% when there is AT heterozygosis in the IFN-γ gene polymorphism at position +874 (HR: 1.49, 95% CI 1.04–2.14), in contrast to patients that have AA homozygosis in the IFN-γ gene.	-
Ocular cytokinome is linked to clinical characteristics in ocular toxoplasmosis	A de-la-Torre et al. (2014) [36]	South America	Case Controls	The total recurrences were positively correlated with the intraocular expression of IL-5 and VEGF.	-
Toxoplasma Serotype Is Associated With Development of Ocular Toxoplasmosis	L Shobab et al. (2013) [38]	Europe	Case Controls	Parasite serotype: • OT patients with a parasite that expresses non-reactive (NR) serotypes were more likely to have recurrences (OR: 2.92; 95% CI 1.05–8.89) (*P* = .037) compared to OT patients with a parasite that expresses other serotypes (Type I/III; Type II; and Atypical) • OT patients with NR serotypes were more likely to have recurrences (OR: 3.42; 95% CI 1.21–10.41) (*P* = .024) compared to OT patients infected with Type II strains.	-
TNF-α gene polymorphism (2308G/A) and toxoplasmic retinochoroiditis	CA Cordeiro et al. (2008) [58]	South America	Case Controls	TNF-α gene polymorphism (2308G/A) does not seem to be associated with the occurrence or recurrence of OT patients.	-
Interleukin-10 Gene Polymorphism (1082G/A) is Associated with Toxoplasmic Retinochoroiditis	CA Cordeiro et al. (2008) [59]	South America	Case Controls	Genotypes related to low production of IL-10 may be associated with the occurrence of OT. However, it was not related with the recurrences and visual acuity.	
Ocular toxoplasmosis: susceptibility concerning the genes encoding the KIR receptors and their HLA class I ligands	CM Ayo et al. (2016) [74]	South America	Case Controls	KIR-HLA inhibitory pair–KIR3DS1-/KIR3DL1+/Bw4-8-80Ile+ combination is a protective factor against recurrent manifestation of OT (OR: 0.13; CI 0.03–0.45)(*P* = 0.0003)	-
*Toxoplasma gondii* in the peripheral blood of patients with acute and chronic toxoplasmosis	C Silveira et al. (2011) [75]	South America	Cross sectional	Parasitaemia could be associated with the reactivation of the ocular disease in immunocompetent individuals.	-
Peptidyl-prolyl cis-trans isomerase A–A novel biomarker of multi-episodic (recurrent) ocular toxoplasmosis	J Isenberg et al. (2018) [77]	North America	Case Controls	One serum protein, peptidyl-prolyl cis-trans isomerase A (PPIA) was confirmed as a biomarker of multi-episodic disease in OT. PPIA can identify the patient with active recurrent OT from acute OT, other forms of uveitis, and other parasitic infections.	-
Interleukin-6 gene polymorphism (-174 G⁄C) is associated with toxoplasmic retinochoroiditis	CA Cordeiro et al. (2013) [86]	South America	Cohort	IL-6 gene polymorphism at position -174 polymorphism was not associated with recurrence (*P* = 0.21)	-

AA: Nitrogenous base adenine/adenine; AT: Nitrogenous base thymine /adenine; CI: Confidence interval; GA: Nitrogenous base guanosine/adenine; IL: Interleukin VEGF: Vascular endothelial growth factor; OR: Odds ratio; OT: Ocular Toxoplasmosis; PPIA: Peptidyl-prolyl cis-trans isomerase A; KIR-HLA: Killer cell immunoglobulin-like receptors- human leukocyte antigen (Part of the KIR gens: KIR3DS1; KIR3DL1; Bw4-8-80Ile); HR: Hazard ratio

**Table 3:** Treatment-related factors influencing reactivations and visual acuity outcomes in OT.

Title	Authors (Year)	Continent	Type of Study	Factors related to recurrences in OT	Factor that influence visual acuity outcome in OT
Long-term Results of Trimethoprim-Sulfamethoxazole Versus Placebo to Reduce the Risk of Recurrent Toxoplasma gondii Retinochoroiditis	Fernandes Felix JP et al. (2020) [9]	South America	Randomized control trial	All the patients (both groups) in the study were treated with TMP-SMZ twice daily for 45 days during the active episode. Then they were randomized to TMP-SMZ every other day for 311 days (group 1) or placebo (group 2). • Significantly higher probability of recurrence was observed in the placebo group compared with the TMP-SMZ group. The cumulative probability of recurrence after 1, 2, 3, 4, 5, and 6 years of follow-up were, respectively, 13.0% (9/69), 17.4% (12/69), 20.3% (14/69), 23.2% (16/69), 26.1% (18/69), and 27.5% (19/69) in the placebo group and 0%, 0%, 0%, 0%, 0%, and 1.4% (1/72) in the TMP-SMZ group (P < .001; log-rank test)	-
Influence of drug therapy on the risk of recurrence of ocular toxoplasmosis	M Reich et al. (2015) [31]	Europe	Cohort	• Median recurrence-free survival time in the subgroup of active lesions treated by *T*. *gondii*-specific antibiotics was 3.0 years (95% CI 2.2 to 3.9 years). -Median recurrence-free survival time in the subgroup treated with systemic corticosteroid monotherapy was 0.9 years (95% CI 0.5 to 1.3 years). • Median recurrence-free survival time in the subgroup without treatment was 2.7 years (95% CI 1.2 to 4.3 years). • Risk of recurrence is significantly higher when treated with systemic corticosteroid monotherapy compared with *T gondii*-specific antibiotic treatment (*P*<0.001) or compared with no therapy (*P* = 0.006). • No significant difference in median recurrence-free survival time between *T*. *gondii*-specific antibiotic treatment versus no therapy was detected (*P* = 0.679).	-
Frequency and factors associated with recurrences of ocular toxoplasmosis in a referral centre in Colombia	A de-la-Torre et al. (2009) [42]	South America	Cross-sectional	Previous therapy with systemic steroids without antibiotics and previous subconjunctival injection of steroids were associated with a higher risk of recurrences (*P* = 0.04, both).	-
A Prospective, Randomized Trial of Pyrimethamine and Azithromycin Vs. Pyrimethamine and Sulfadiazine for the Treatment of Ocular Toxoplasmosis	LH Bosch-Driessen et al. (2002) [45]	Europe	Randomized control trial	Group 1: Pyrimethamine and Azithromycin VS Group 2: Pyrimethamine and Sulfadiazine During the first year after treatment, recurrences occurred in 5 out of 15 patients (33%) in group 1, and 5 out of 9 patients (56%) in group 2.	-
Ocular Toxoplasmosis Clinical Features and Prognosis of 154 Patients	LEH Bosch-Driessen et al. (2002) [7]	Europe	Cross-sectional	-	• Legal blindness was more common in patients who had ever been treated with corticosteroids without antiparasitic drugs compared to those who received other or no treatments (*P* = 0.0004)
The Impact of Short-Term, Intensive Antifolate Treatment (with Pyrimethamine and Sulfadoxine) and Antibiotics Followed by Long-Term, Secondary Antifolate Prophylaxis on the Rate of Toxoplasmic Retinochoroiditis Recurrence	PK Borkowski et al. (2016) [11]	Europe	Cross-sectional	• All patients received treatment during the first 21 days with different schemes (pyrimethamine/ sulfadoxine or spiramycin, azithromycin, and steroid). The secondary antifolate prophylaxis (A-SP) was instituted immediately after the patient completed the first phase of treatment (pyrimethamine/sulfadoxine 25 mg/500 mg, one tablet twice a week for six months without folinic acid supplementation). • This secondary scheme shows a 3-year recurrence-free survival after the first course of A-SP in 90.9% of cases. • With this secondary scheme, the pre-existing retinal scars have RR 2.41 95% IC 1.14–6.95 (*P* = 0.02) risk of a recurrence. Nonetheless, this effect was lost in the multivariate analysis aRR 2.41 95% IC 0.96–6.04 (*P* = 0.06) • In the Kaplan-Meier was evidenced that the probability of recurrence was lower in patients without a pre-existing scar (*P* = 0.075) and in patients with retinal hemorrhage (*P* = 0.033)	-
The Effect of Long-term Intermittent Trimethoprim/Sulfamethoxazole Treatment on Recurrences of Toxoplasmic Retinochoroiditis	C Silveira et al. (2002) [53]	South America	Randomized control trial	The HR for the recurrences was 0.25 (95% CI 0.08–0.75) in favor of the treated group, indicating a 75% reduction in recurrences.	
Laser Photocoagulation around the Foci of Toxoplasma Retinochoroiditis: A Descriptive Statistical Analysis of 35 Patients with Long-Term Follow-Up	T. Desmettre et al. (1996) [60]	Europe	Cross-sectional	Laser photocoagulation around the lesion: Recurrence rates with 95% IC were: 1 year (12.7±13%); at 2 years (19.8±15%); at 3 years (24.0±16%); at 4 years (33.3± 19%); at 5, 6 and 7 years (53.5±21%); at 8 and 9 years (66.8±22%). With the data, it is impossible to show the efficacy of laser photocoagulation in the prevention of recurrence in toxoplasmic retinochoroiditis.	
Ocular Toxoplasmosis–seeking a strategy for treatment	Prášil et al. (2014) [61] (PMID: 25702053)	Europe	Cross-sectional	Scheme: Pyrimethamine/Sulfadiazine+Steroid: 5 Recurrences post treatment. Scheme: Azithromycin+Steroid: All had seven recurrences post-treatment. Other schemes: All had five recurrences post-treatment. Azithromycin scheme evidence a higher rate of recurrence.	
Efficacy of specific chemotherapy in the prevention of recurrences of toxoplasmic chorioretinitis during the 4 years following the treatment	J.C. Timsit et al. (1987) [5]	Europe	Non-randomized controlled study	Pyrimethamine-Sulfadiazine therapy appears to be an effective synergistic therapy to prevent recurrent toxoplasmosis uveitis.	
Ocular Outcome of Brazilian Patients With Congenital Toxoplasmosis	EG Lago et al. (2021) [73]	South America	Cohort	The mean duration of treatment for congenital toxoplasmosis within the first year of life was higher in patients without retinochoroiditis development or recurrence (9.6±2.8 months) than in those patients who developed new lesions (7.7±3.8 months) (*P* = 0.01).	-
Prophylactic Photocoagulation of Recurrent Toxoplasmic Retinochoroiditis	HF Spalter et al. (1966) [81]	North America	Cohort	Photocoagulation after treatment shows that none of the 24 patients have developed recurrent retinitis in the photocoagulation areas during a 6- to 33-month follow-up period.	-
Ocular Toxoplasmosis in Human Immunodeficiency Virus-infected Patients	I Cochereau-Massin et al. (1992) [84]	Europe	Cross-sectional	The 24-month relapse rates were 0.20 and 0.18 for the 50-mg/day and 25-mg/day dosage of pyrimethamine, respectively. The overall 12-month survival rate was 0.72.	-
Management of Severe Ocular Toxoplasmosis with Intravitreal Clindamycin and Triamcinolone: Report of 22 cases.	H Ocampo Dominguez (2015) [72]	South America	Cohort	-	Visual acuity expressed in LogMAR changed after treatment, from 1.05 before treatment to 0.51 (*P* = 0.002) using intravitreal clindamycin.
Comparison of Azithromycin and Pyrimethamine/ Sulfadiazine Treatment in Ocular Toxoplasmosis in North West of Iran	LA Ghavide et al. (2017) [71]	Asia	Randomized control trial	Group 1: Pyrimethamine and Sulfadiazine vs. Group 2: Azithromycin Recurrences were observed in 4/36 (11.1%) patients in Group 1 and 18/36 (50%) in Group 2.	-
A prospective randomized trial of azithromycin versus trimethoprim/ sulfamethoxazole in treatment of toxoplasmic retinochoroiditis	A Lashay et al (2016) [87]	Asia	Randomized control trial	-	Group 1: Azithromycin vs. Group 2: trimethoprim/sulfamethoxazole. There was a significant improvement in VA after treatment in both groups; it improved by 0.24 ± 0.04 LogMAR in the azithromycin group (*P* = 0.001) and by 0.30 ± 0.01 LogMAR in the trimethoprim/sulfamethoxazole group (*P* = 0.001). There was no statistically significant difference between groups (*P =* 0.17)
Toxoplasmose ocular: estudo comparativo do tratamento com sulfadiazina e pirimetamina versus sulfametoxazol e trimetropim / Ocular toxoplasmosis: a comparative study of the treatment with sulfadiazine and pyrimethamine versus sulphametoxazole-trimethoprim	E Raskin et al. (2002) [89]	South America	Non-randomised controlled study	-	Therapeutic factor: G1: Pyrimethamine and Sulfadiazine VS G2: TMP/SMX No difference was found in the mean VA difference post-treatment.

BCVA: Best corrected visual acuity; CI: Confidence intervals; DD: Disc of diameter; VA: Visual acuity; OT: Ocular Toxoplasmosis; RR: Relative Risk; OR: Odds Ratio; HR: Hazard ratio

### Clinical and environmental factors

Multiple articles described clinical factors that influence the recurrence rate. One of them was time since the disease onset. Most authors found a higher risk of reactivation in the first years after primary acquired infection, which tends to decrease over time. This risk can reduce by 72% in the first decade, and the decrease is continuous over time [14, 44, 76, 78]. In addition, age over 40 years was a factor that increased the risk of recurrences with a relative risk (RR) up to 1.74, (95% CI 1.06–2.86) [44]. Other clinical factors increasing the risk of OT reactivation were bilateral lesions (RR: 8.0; 95% CI 1.3–50.0) [28], pregnancy (RR: 7.40; 95% CI 4.46–12.06) [33], the presence of a single lesion with a Hazard Ratio (HR) 1.60 (95% CI 1.07–2.40) [12], and an initial active presentation odds ratio (OR) 4.74 (95% CI 1.95–12.91) [27, 42].

On the other hand, environmental factors, such as living in regions with high precipitation rates, were also associated with higher recurrence rates. An Argentinian study found that for every mm of precipitation, the frequency of consultations due to OT recurrences increased by 2% in an ophthalmological center (OR: 1.002; 95% CI 1.000–1.003) [54]. Other factor is the consumption of bottled water that reduces recurrences risk [34]; on the other hand, surgeries are controversial as risk factor of reactivation [46, 47].

Regarding recurrences in previous and no previous involved eye, they usually occur in the affected eye [7, 85]. Finally, the mean number of reactivations was between 0.105 and 2.6 recurrences per year [14, 28, 31, 50]. In addition, reported flare-ups per interval of time were one recurrence in 5 years, 1.7 recurrences in 10 years, and two recurrences in 11 years [42, 44, 50]. However, it is not possible to meta-analyze this information due to the data heterogeneity.

Concerning visual impairment, studies have found that patients > 45 years of age who debut with active lesions have a ten times higher risk of developing visual impairment [27, 32, 79]. Moreover, the location of the lesion also plays an essential role, as the involvement of the macular area is associated with 8.95 times more occurrence of visual impairment than the peripheral retinal compromise [27, 32, 37, 65]. Also, complications (e.g. retinal detachment) are important factors influencing visual outcomes (OR: 10.26; 95% CI 3.82–30.67) [6, 27]. Other clinical factors related to visual impairment are the grade of inflammation, and lesion size [65, 70, 79]. Congenital Toxoplasmosis increased the risk of visual impairment (RR: 5.07; 95% CI 2.62–9.81) [7, 57, 66]; however, in these patients visual impairment is also determined by macular localization (RR: 3.06; 95% CI 1.16–9.28) [66].

The development of blindness, as reactivations and visual impairment, was affected by the location of the lesion. It was estimated that central lesions raise 8.95 to 68.6 times the risk of blindness compared to a lesion located outside the macula [7, 27, 35, 37, 64]. Additionally, an age ≥50 years and the presence of lesions size >1 disc diameter (DD) had an OR of 3.27 (95% CI 1.22–8.8) and 6.30 (95% CI 2.28–22.46) for blindness, respectively [7, 27, 64]. Other associated factors are a history of scarring, recurrences during follow-up, and a VA <20/200 at admission [32, 35, 64]. Interestingly, in one article the geographical region was not statistically significantly associated with reactivations, visual impairment, and blindness [64]. Finally, we could consider some variables as future biomarkers, such as the evidence of hyporeflective spaces or signal voids in Spectral Domain optical coherence tomography [52]. Nevertheless, in OT patients the standard automated perimetry better indicate the moderate or severe visual impairment (blindness) than visual acuity [51].

### Parasite and host factors influencing reactivations and visual acuity outcomes in OT

Factors related to the parasite can influence OT recurrences, L Shobab et al. found that patients infected with serotypes non-reactive (NR) to GRA6 and GRA7 allelic peptide motifs derived from distinct parasite types were more likely to have recurrences (OR: 2. 92; 95% CI, 1.05–8.89) compared to OT patients infected with a type I/III, type II, or atypical parasites. Moreover, they described that patients with NR serotype had 2.42 times more recurrences than those infected with type II strains [38].

On the other hand, there are protective and risk factors related to the human host. The AT heterozygosis polymorphisms in the IFN-γ gene at position +874 increases the risk of recurrence by 49% (HR: 1.49, 95% CI 1.04–2.14) [12]. Additionally, other genetic studies have found that genotypes associated with low production of IL-10 may be associated with the OT development but not with the recurrence and visual acuity [59]. Likewise, the TNF-α gene polymorphism (2308G/A) do not seem to be associated with OT development and recurrences [58]. Similarly, Ayo et al. found that the presence of the -KIR3DS1/KIR3DL1+/Bw4-8-80Ile+ combination was a protective factor against recurrences (OR: 0.13; 95% CI 0.03–0.45) [74].

Finally, a study involving intraocular fluids have found that elevated levels of IL-5 and VEGF positively correlated with recurrences [36]. Also, Silveira C et al. proposed that parasitemia could be a factor related to reactivations, based on the analysis of peripheral blood mononuclear cells (PMBC); however, the sample in this study was small limitating the results generalization [75]. Finally, peptidyl-prolyl cis-trans isomerase A (PPIA) identified by immunoblotting may be a biomarker of multi-episodic disease in OT patients. PPIA can help deferring between a first episode of OT, a recurrence of OT, other forms of uveitis, or other parasitic infections during an active ocular inflammation episode [77].

### Treatment-related factors influencing reactivations and visual acuity outcomes in OT

Several treatment regimens used in active OT seem to influence the risk of recurrences, such as trimethoprim-sulfamethoxazole (TMP/SMX), spiramycin (Sp), and pyrimethamine sulfadiazine (P+Sdz), in combination with or without systemic and topical steroids. However, studies have not shown that treatment in the disease’s active phase can significantly reduce the recurrence rate. Prospective studies are needed to analyze this outcome in more detail [5, 9, 31, 45, 61, 71]. However, some studies debate this; proposing the use of antibiotic therapy is not superior to the use of no management at all during active episodes of OT [90, 91]. This issue still needs more studies with optimal methodological quality [31]. Additionally, the use of systemic steroids without antibiotic treatment and subconjunctival steroids are a determining factors for the presence of new recurrences [42].

On the other hand, studies have shown that the regimen of azithromycin alone or with a steroid has less effectiveness than other schemes [61, 71], achieving comparable effectiveness only when combined with pyrimethamine [45]. However, some of these studies have a poor methodological quality, so their conclusions do not have a high validity. Therefore, the information regarding the use of azithromycin for recurrences should be analyze cautiously (see **Fig 3A and 3B and S2 Table**).

The use of pyrimethamine/sulfadoxine (Sdx) as secondary antifolate prophylaxis can lead to 3,5-year recurrence-free survival in 90.9% of cases (Scheme: P+Sdx 25 mg/500 mg, one tablet twice a week for six months without folinic acid supplementation) [11]. Another proposed scheme of prophylaxis is the single tablet TMP160 mg/SMX 800 mg every three days for up to 20 months, showing a 75% reduction of recurrences with a HR of 0.25 (95% CI, 0.08–0.75) [53]. Also, another scheme with TMP/SMX was described by Fernandes Felix et al. where they gave one dose of TMP160 mg/SMZ 800 every other day for 311 days, showing a single recurrence in 6 years in the experimental group [9]. Moreover, two studies report the use of photocoagulation for the prevention of reactivation; however, these studies do not have much methodological soundness and contradict each other [60, 81]. Additionally, in immunosuppressed patients, the prophylactic scheme with P+Sdz seems to reduce the rate of recurrences, however, more studies are needed [84].

Regarding VA, a significant effect of antibiotic therapy is observed in general, but more studies of better quality are needed to make any affirmation [43, 45, 72, 87, 89].

### Meta-analysis

#### Prevalence of recurrences in OT patients

**Fig 4:**
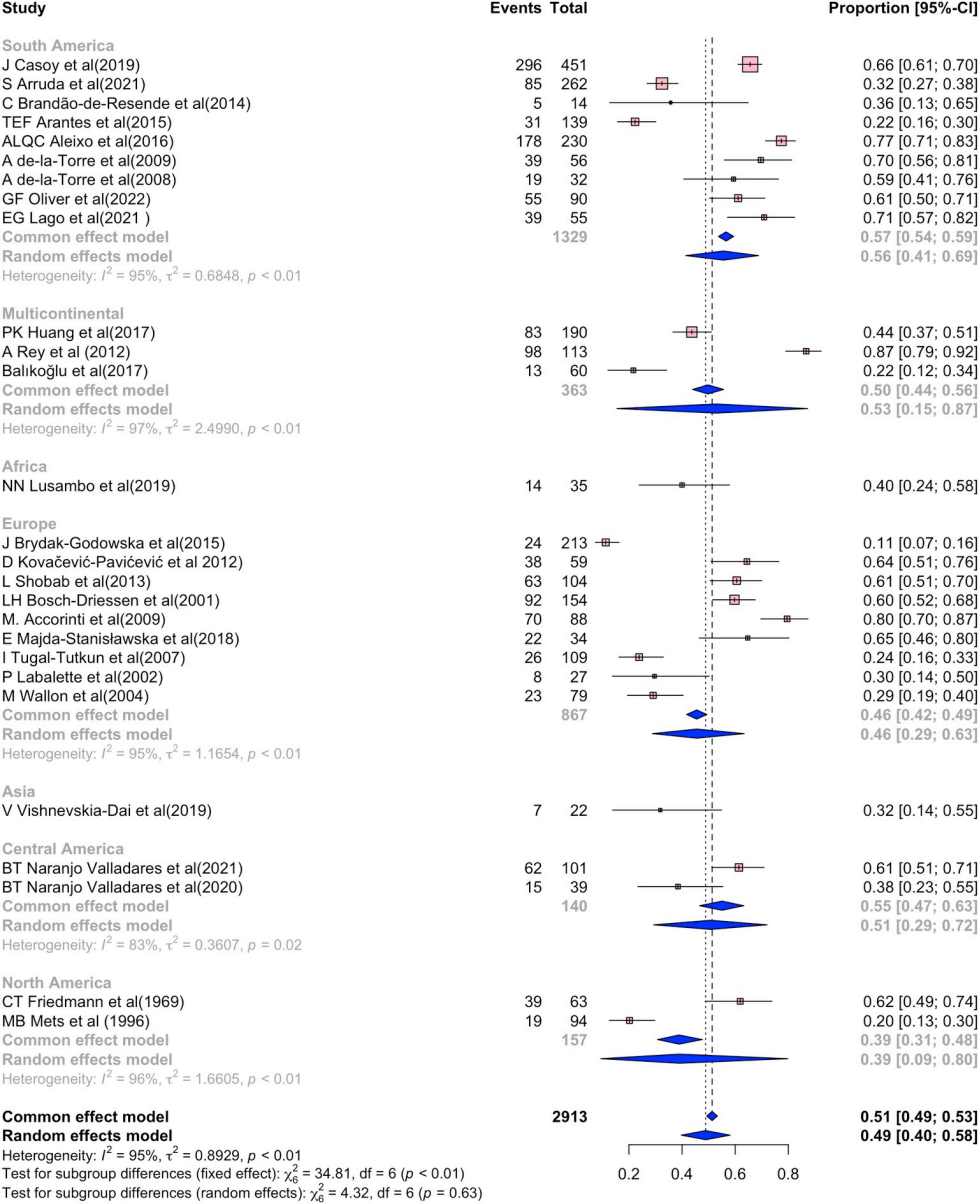
Frequency of recurrence segmented by continent.

In the meta-analysis for the prevalence recurrences, 2,913 patients with OT were included (Fig 4), and the global prevalence of recurrences was 49% (95% CI 40%–58% I2 = 95%; Fig 4) (*P* < 0.01). Given the demonstrated high level of heterogeneity, an analysis by continental subgroups was performed, where the p-value lost statistical significance (*P* = 0.63). The highest prevalence was reported in South America at 56% (95% CI 41%–69% I2 = 95%; Fig 4), followed by Central America at 51% (95% CI 29%–72% I2 = 83%), Europe 46% (95% CI 29%–63% I2 = 95%; Fig 4), and North America 39% (95% CI 9%–80% I2 = 96%; Fig 4) [7, 26–30, 32, 35, 37, 38, 40, 42, 48, 52, 55, 63, 64, 68–70, 73, 78, 80, 83, 85]. Funnel plot of all studies evidences a Fail-Safe N analysis (Fail-safe N = 32976; P<0.001), Rank correlation test (Tau = -0.058; P = 0.681), Asymetry (Z = -0.353; P = 0.724) can be found in the **S1 Fig**.

#### Prevalence of visual impairment and blindness in OT patients

**Fig 5:**
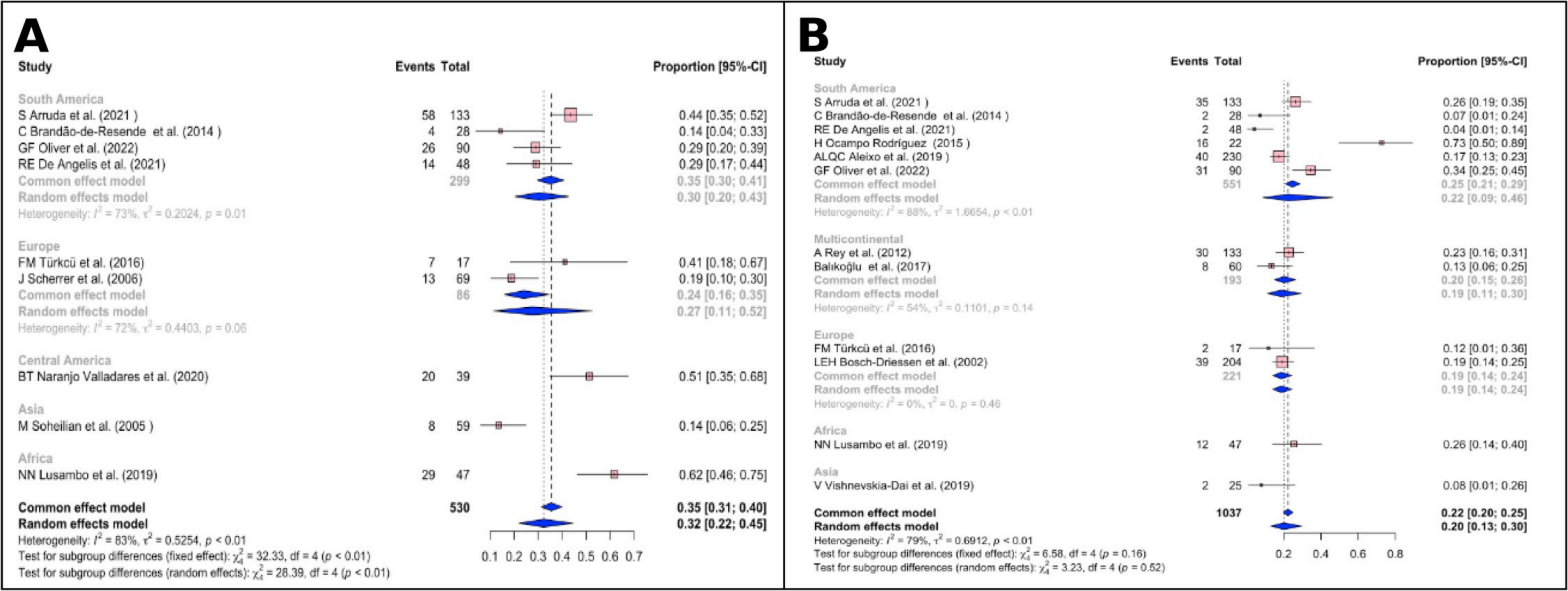
Frequency of visual impairment and blindness of OT. (A).Visual impairment, (B). Blindness.

Four hundred seventy one eyes were analyzed to estimate the prevalence of visual impairment secondary to OT (Fig 5A). This was found at 35% (95% CI 25% –48%, I 2 = 80%; (*P* <0.01) Fig 5A) of cases around the world. Subgroup analysis shows that the continent with the highest prevalence of visual impairment was South America with 30% of cases (95% CI 20%–43%, I 2 = 73%; (*P* = 0.01)), followed by Europe 27% (95% CI 11%–52%, I 2 = 72%; (*P* = 0.06)) [25, 27, 28, 32, 51, 52, 69, 88]. Funnel plot of all studies showed a Fail-Safe N analysis (Fail-safe N = 857; P <0.001), Rank correlation test (Tau = 0.333; P = 0.260), Asymetry (Z = 1.201; P = 0.230) (**S1 Fig)**.

Regarding the prevalence of blindness in OT (Fig 5B), 969 eyes were analyzed and it was 20% (95% CI 13%–30%, I 2 = 79%; (*P* <0.01)). Subgroup analysis showed the frequency of blindness was slightly lower in Europe 19% (95% CI 14%–24%, I 2 = 30%; (*P* = 0.46)) than in South America 22% (95% CI 9%–46%, I2 = 88% (*P* <0.001)). In addition, in visual impairment even performing a subgroup analysis the statistically significant difference between studies remains [7, 12, 25, 27, 28, 32, 40, 52, 64, 68, 72, 88]. Funnel plot of all the studies can be found in the **S1 Fig**, the Fail-Safe N analysis (Fail-safe N = 1106; P <0.001), Rank correlation test (Tau = 0.182; P = 0.459), Asymetry (Z = 1.620; P = 0.105).

#### Factors influencing recurrences

**Fig 6:**
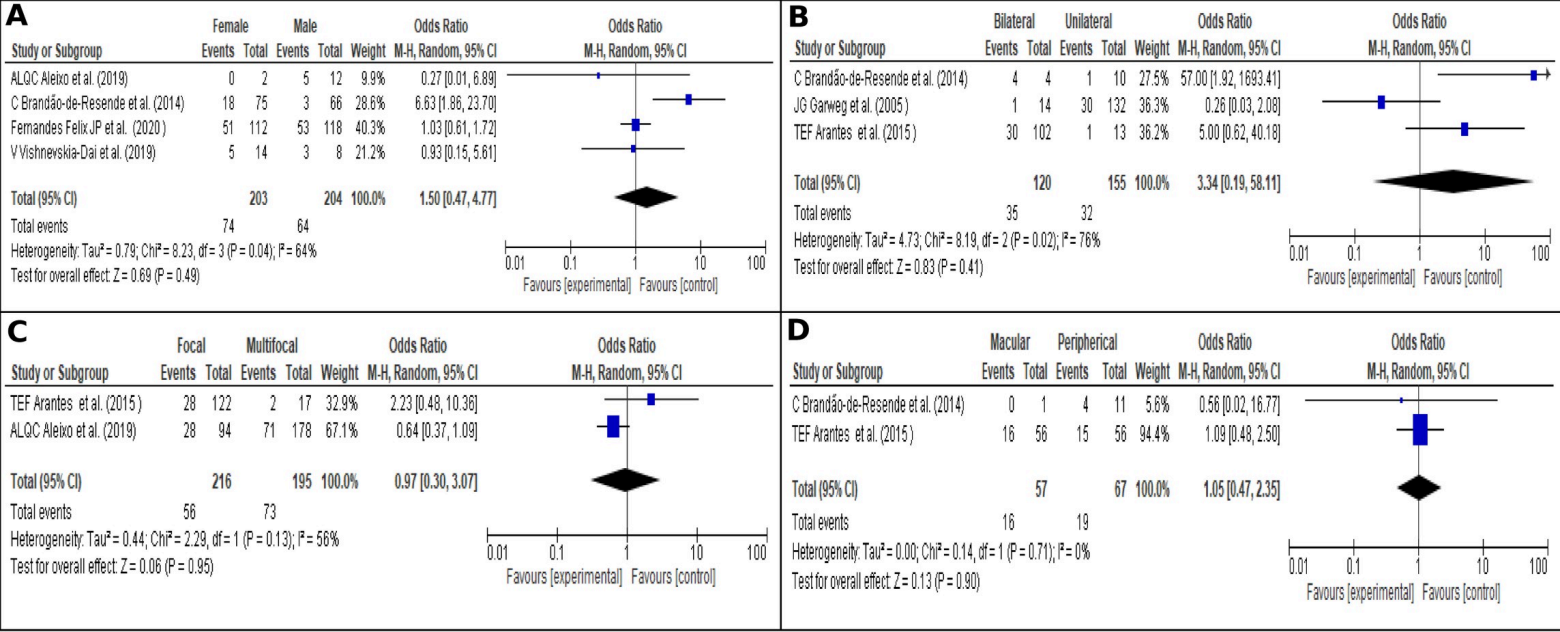
Recurrences related factors. (A). Laterality, (B). Localization; (C). Sex; (D). Number of lesions.

None of the factors evaluated was significantly associated with recurrences. However, some tendencies were found. Fig 6A shows the effect of gender in recurrences, OR 1.50 (95% CI; 0.47–4.77; I2 = 64% (*P* = 0.49)) [9, 12, 28, 40]. Fig 6B evidences the effect of laterality in recurrences, OR 3.34 (95% CI; 0.19–58.11; I2 = 76% (*P* = 0.41)) [28, 30, 82]. Fig 6C shows the effect of the number of lesions in recurrences, OR 0.97 (95% CI; 0.30–3.07; I2 = 56% (*P* = 0.95)) [12, 30]. Fig 6D shows the effect of the lesion localization in recurrences, OR 1.05 (95% CI; 0.47–2.35; I2 = 0% (*P* = 0.90)) [28, 30].

#### Blindness related factors

**Fig 7:**
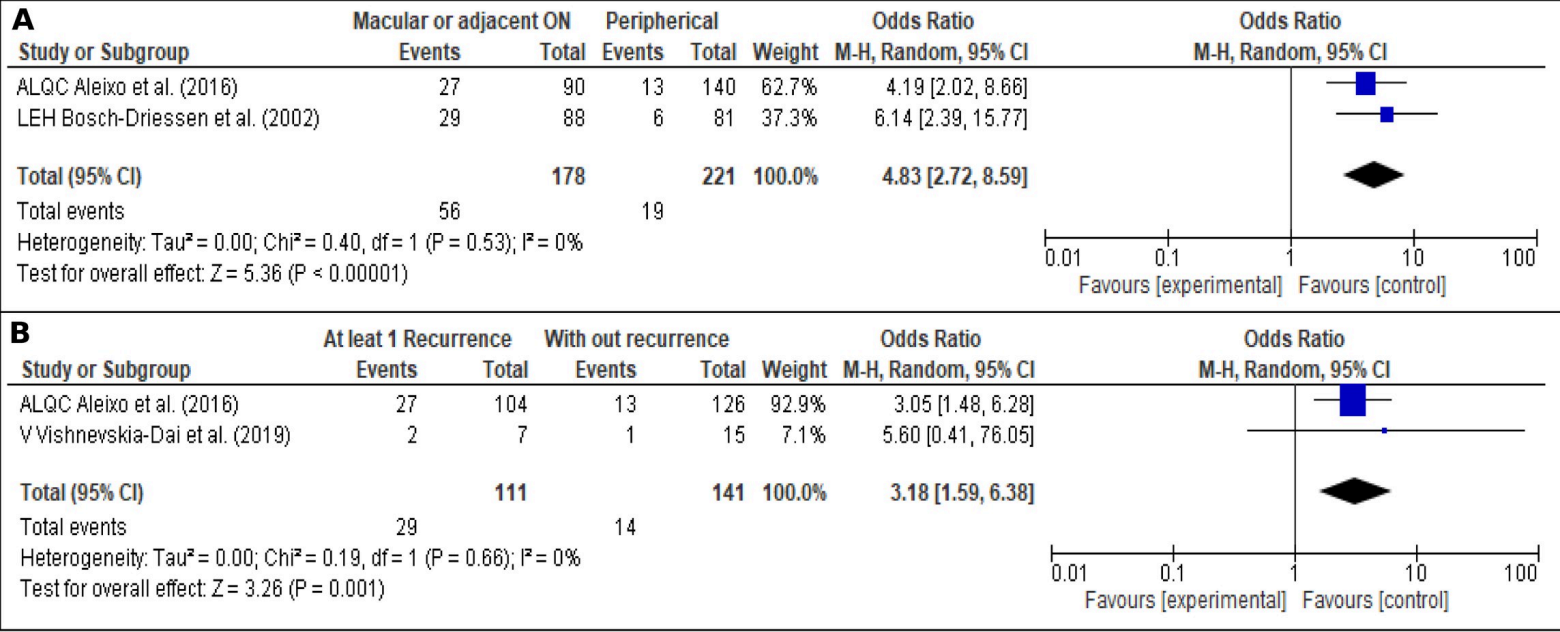
Blindness related factors. (A). Localization, (B). Recurrences.

We evaluated the factors related to blindness in OT patients. Fig 7A shows that macular or adjacent optic nerve lesions are significantly related to blindness, OR 4.83 (95% CI; 2.72–8.59; I2 = 0% (*P* <0.00001)) [7, 35]. Also, Fig 7B, shows that recurrences are a risk factor for blindness, OR 3.18 (95% CI; 1.59–6.38; I2 = 0% (*P* = 0.001)) [35, 40].

#### Effect of the initial treatment in the reduction of recurrences

**Fig 8:**
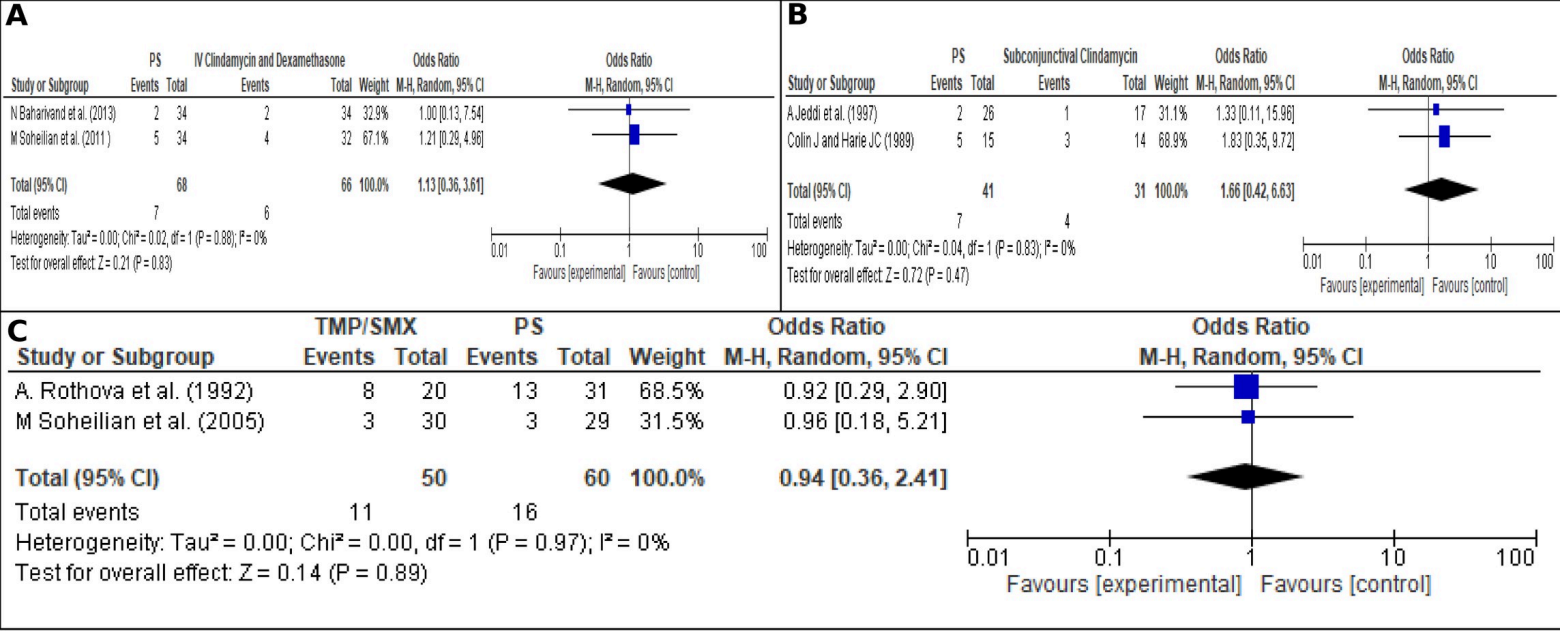
Effect of the treatment on the recurrences and visual factor. (A). Any scheme of P+Sdz vs Intravitreal clindamycin + dimethazone, (B). Any scheme of P+Sdz vs Subconjunctival clindamycin, (C). Any scheme of TMP/SMX vs Any scheme of P+Sdz.

We sought to determine if any treatment further reduced recurrences, none of them were significantly associated with a reduction on recurrences. Fig 8A shows the comparison between any P+Sdz scheme vs. the use of intravitreal clindamycin + dexamethasone, OR 1.13 (95% CI; 0.36–3.61; I2 = 0% (*P* = 0.83)) [39, 56]), Fig 8B shows the comparison between any P+Sdz scheme vs. subconjunctival clindamycin, OR 1.66 (95% CI; 0.42–6.63; I2 = 0% (*P* = 0.47)) [62, 67]. Fig 8C compares the use of any TMP/SMX scheme vs P+Sdz scheme OR 0.94 (95% CI; 0.36–2.41; I2 = 0% (*P* = 0.89) [41, 43]. In this analysis, risk trends are observed, without significance, where clindamycin by any ocular route of administration reduces recurrences more frequently than any P+Sdz scheme, and TMP/SMX also showed to be partially better for reducing recurrences vs. P+Sdz.

#### Prophylaxis treatment to reduce recurrence

**Fig 9:**
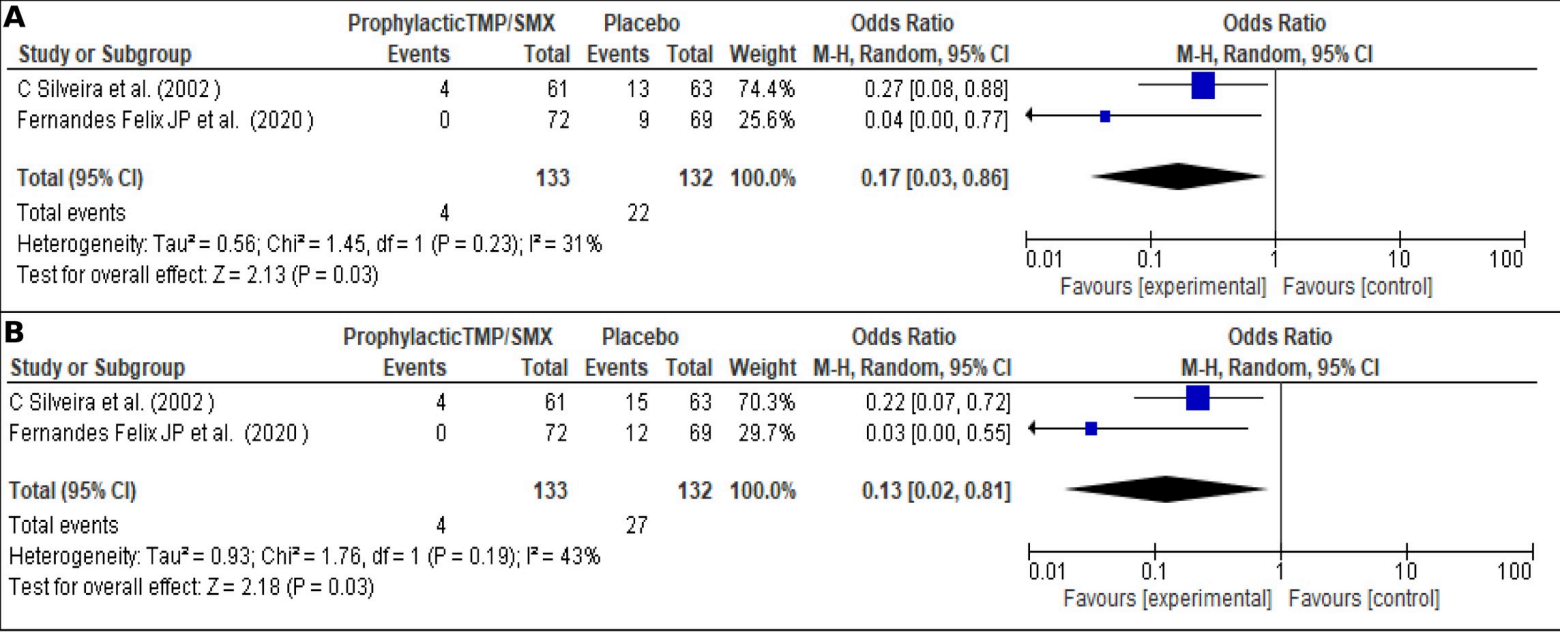
Effect of the prophylaxis on the recurrences. (A). Reduction in recurrence with prophylaxis in the first year, (B). Reduction in recurrence with prophylaxis in the second year.

This analysis sought to determine the effect of prophylactic treatments reducing recurrences [9, 53]. Fig 9A, shows the effect at the first year after the TMP/SMX prophylaxis vs placebo OR 0.17 (95% CI; 0.03–0.86; I2 = 31% (*P* = 0.03)). Fig 9B shows the effect at the second year after the TMP/SMX prophylaxis vs placebo, 0.13 (95% CI; 0.02–0.81; I2 = 43% (*P* = 0.03)). In this case, a statistical significant result was evidenced, also, a tendency of reduction in the risk of recurrence was incremental over time.

## Discussion

Our systematic review and meta-analysis evaluated the effect of multiple clinical, environmental, geographic, parasite, genetic, and management factors on the recurrences, visual impairment, and blindness in OT.

One of the most critical factors related to recurrences was the first year after the first lesion because the risk tends to decrease in the subsequent years [7, 14]. Additionally, age >40 years, binocularity of the lesions, pregnancy, precipitation, parasite with a non-reactive serotype, AT heterozygosis in the IFN-γ gene at position +874, and the use of steroids without antibiotics as treatment were the main determinants of reactivations. The majority of these factors can be explained by the the immunosenescence that allows reactivations of the parasite cyst located in the retina [12, 14, 27, 44]. Therefore, these are the variables that should be considered when elaborating additional recommendations [14, 28, 38, 42, 44, 54].

Additionally, we meta-analyzed the prevalence of recurrences, visual impairment, and blindness worldwide. We found that recurrences are most common in South American and Central American patients, corroborating that these latitudes have more severe cases of OT, possibly due to more virulent strains [92, 93]. However, all studies in Central America evaluated only one region (Cuba), which can generate an overestimation of the effect. On the other hand, it should be considered that the meta-analysis of prevalence of recurrence has a high risk of publication bias, which is why they should be analyzed carefully, and the subgroup’s analysis did not entirely correct them. Owing to this, we suggest that there are additional factors that explain the high heterogeneity. For example, the serotype could be another factor, as L Shobab et al. [38] reported that the NR serotype had the highest risk of recurrence, which may be considered in future studies.

Regarding visual acuity and blindness prevalence in OT eyes, we found that in South America there is a greater prevalence of visual impairment and blindness than in Europe but not in the rest of the world. However, some studies’ incorrect reports of this outcome can limit our results. Additionally, it should be noted that the sub-analysis by subgroups appropriately controlled the heterogeneity of the metanalysis.

In the analysis of risk factors associated with blindness, we found that recurrences and the macular localization of the lesion are the most important factors related to blindness. This has been supported by previous studies, which evidenced that macular lesions have up to 8.95 to 68.6 times increased risk of blindness [7, 27, 35, 37, 64]. Therefore, as clinicians, we have to prevent these scenarios to reduce the visual impact of OT.

In addition to the previously mentioned factors (macular location of the lesions and recurrences) [7, 27, 32, 35, 37, 64, 65], other factors that increased the risk of blindness are extensive or atypical lesions in patients aged more than 45 years [7, 27, 32, 64, 79], and congenital toxoplasmosis [7, 57, 66]. Therefore, a closer follow-up and optimized treatment should be considered in these patients. Moreover, we evidence that few articles detail the immunological status of patients, which should be added in future studies in order to quantify the impact of this variable. Regarding the treatment in active lessons, we found that TMP/SMX and clindamycin (intravitreal and subconjunctival) are possibly superior to PS in preventing recurrence; nevertheless, these differences were not statiscally significant and must be interpreted with caution. Prospective studies are needed to evaluate the best therapy in the acute phase of the disease that further prevents recurrences.. Although there is a lack of data, current evidence can be useful to guide prophylactic treatment [39, 43, 56, 62, 67].

Prophylaxis in patients with OT is a protective factor that increases over time, reaching a reduction of almost 87% compared to placebo. This metanalysis does not have a high risk of publication bias. Also, it is essential to consider there were evaluated few studies; we suggest conducting more studies in this field to expand the evidence and assess the superiority of the different schemes. Our results are supported by the marked reduction trend in recurrences that has been reported with the use of antibiotic prophylaxis with TMP/SMX in any scheme, more detailed information on schemes, doses and times see **Table 4** [9, 53]. Nevertheless, a publication by Silveira et al. showed after discontinuing the TMP/SMX treatment, the recurrence rate is similar to the placebo group, even after ten years of use. This is explained due to a loss of the drug’s effect once discontinued [8]. This differs from Fernandes Felix et al., who have shown a much lower recurrence rate after the discontinuation of the prophylactic scheme, with six years free of recurrences [9]. We proposed that other possible variables could be involved, such as the periodicity of the prophylactic treatment, parasite genotype, genetic susceptibility of the host, and the immune status, that may differ from the studies’ populations [9, 11, 53]. We suggest performing further studies on these additional variables.

**Table 4:** Prophylaxis scheme in OT.

Prophylactic therapy to avoid recurrences: In patients with OT it refers to an additional scheme of treatment during at least 6 months. However, schemes and times vary between articles [9, 11, 53].				
Title	Authors (Year)	Initial antibiotic treatment	Prophylactic therapy	Impact of prophylaxis
Long-term Results of Trimethoprim-Sulfamethoxazole Versus Placebo to Reduce the Risk of Recurrent Toxoplasma gondii Retinochoroiditis	Fernandes Felix JP et al. (2020) [9]	All the patients in the study were treated with TMP-SMZ (800 mg/160 mg) twice daily for 45 days. Recurrences were treated with 1 tablet of TMP-SMZ (800 mg/160 mg) twice daily for 45 days.	Then they were randomized to TMP-SMZ every other day for 311 days (group 1) or placebo (group 2). Group 1: 1 dose of TMP-SMZ (800 mg/160 mg) every other day for 311 days. Group 2: Placebo.	The cumulative probability of recurrence after 1, 2, 3, 4, 5, and 6 years of follow-up were, respectively, 13.0% (9/69), 17.4% (12/69), 20.3% (14/69), 23.2% (16/69), 26.1% (18/69), and 27.5% (19/69) in the placebo group and 0%, 0%, 0%, 0%, 0%, and 1.4% (1/72) in the TMP-SMZ group (P < .001; log-rank test)
The Impact of Short-Term, Intensive Antifolate Treatment (with Pyrimethamine and Sulfadoxine) and Antibiotics Followed by Long-Term, Secondary Antifolate Prophylaxis on the Rate of Toxoplasmic Retinochoroiditis Recurrence	PK Borkowski et al. (2016) [11]	All the patients received P/S (50 mg/ 1,000 mg daily for the first two days, followed by P/S(25 mg / 500 mg) on days 3–21, one tablet daily. Additionally, starting from day 2 spiramycin 3 million IU three times daily was administered for 10 days followed by azithromycin 0.5 g once daily for another 6 days. Folinic acid was not routinely supplemented. (Just in cases of thrombocytopenia, until their platelet counts returned to normal). All patients in the day 2 received oral prednisone, usually 40 mg in the morning. (Gradually tapered over 4–6 weeks depending on the resolution of the inflammation and associated exudate).	All the patients received O/S (25 mg/500 mg) one tablet twice a week for 6 months without folinic acid supplementation.	• 3-year recurrence-free survival after the first course of A-SP in 90.9% of cases. • With this secondary scheme, the pre-existing retinal scars have RR 2.41 (95% IC 1.14–6.95) risk of a recurrence, but it was lost in the multivariate analysis aRR 2.41 (95% IC 0.96–6.04) (*P* = 0.06) • In the Kaplan-Meier was evidenced that the probability of recurrence was lower in patients without a pre-existing scar (*P* = 0.075) and in patients with retinal hemorrhage (*P* = 0.033).
The Effect of Long-term Intermittent Trimethoprim/Sulfamethoxazole Treatment on Recurrences of Toxoplasmic Retinochoroiditis	C Silveira et al. (2002) [53]	-	Adults: Treated patients received a single tablet of a commercially available combination of TMP-SMZ (800 mg/160) every 3 days for up to 20 months (the planned duration of the study). Children: A liquid suspension of trimethoprim (40 mg/5ml)/sulfamethoxazole (200 mg/5ml) at a dose of 0.375 ml/kg orally every 3 days.	The HR for the recurrences was 0.25 (95% CI 0.08–0.75).

TMP-SMZ: Trimethoprim/Sulfamethoxazole; P/S: Pyrimethamine /Sulfadoxine; CI: Confidence intervals; OT: Ocular Toxoplasmosis; RR: Relative Risk; HR: Hazard ratio.

In conclusion, considering the results of our meta-analysis and the evidence previously presented, we suggest that patients older than 40 years, patients with de novo OT lesions or with less than one year after the first episode, macular area involvement, lesions greater than 1DD, congenital toxoplasmosis, and bilateral compromise could benefit from using prophylactic therapy, especially if they live in South America or in a high precipitation area. However, due to the number of studies and their characteristics, it is not possible to determine which prophylactic strategy is superior [9, 11, 53].

## Supporting information

S1 Checklist PRISMA 2020 checklist. (DOCX)

S1 Table Search strategy. (DOCX)

S2 Table Risk of bias and quality assessment. (DOCX)

S3 Table Characteristics of the metanalyzed studies. (DOCX)

S1 Fig Funnel-plots. Fail-Safe N analysis (Fail-safe N = 30630; P<0.001), Rank correlation test (Tau = -0.066; P = 0.650), Asymmetry (Z = -0.375; P = 0.708). Fail-Safe N analysis (Fail-safe N = 857; *P* <0.001), Rank correlation test (Tau = 0.333; *P* = 0.260), Asymmetry (Z = 1.201; *P* = 0.230). Fail-Safe N analysis (Fail-safe N = 1086; *P* <0.001), Rank correlation test (Tau = 0.212; *P* = 0.381), Asymmetry (Z = 2.225; *P* = 0.026).
(DOCX)

## Abbreviations

AA: Nitrogenous base adenine/adenine
AT: Nitrogenous base thymine /adenine
BCVA: Best corrected visual acuity
CI: Confidence interval
DD: Disc of diameter
GA: Nitrogenous base guanosine/adenine
HR: Hazard ratio
IL: Interleukin
KIR-HLA: Killer cell immunoglobulin-like receptors- human leukocyte antigen (Part of the KIR gens: KIR3DS1; KIR3DL1; Bw4-8-80Ile)
OR: Odds Ratio
OT: Ocular Toxoplasmosis
PPIA: Peptidyl-prolyl cis-trans isomerase A
RR: Relative Risk
VA: Visual acuity
VEGF: Vascular endothelial growth factor
Sp: Spiramycin
P+Sdz: Pyrimethamine Sulfadiazine
P+ Sdx: Pyrimethamine Sulfadoxine
AO: Álvaro Olate Pérez
CCG: Carlos Cifuentes-González
EC: Érika Carvalho
FV: Felipe Valenzuela
LM: Lucía Miguel-Escuder
MS: María Soledad Ormaechea
MH: Milagros Heredia
PB: Pablo Baquero-Ospina
WR: William Rojas-Carabali
AA: Alfredo Adan
AC: Andre Curi
AS: Ariel Schlaen
CU: Cristhian Alejandro Urzua
CC: Cristóbal Couto
LA: Lourdes Arellanes
ADLT: Alejandra de-la-Torre
