# Supplementary material for: Risk factors for recurrences and visual impairment in patients with ocular toxoplasmosis: A systematic review and meta-analysis
Source: PLoS One. 2023 Apr 3;18(4):e0283845. doi: 10.1371/journal.pone.0283845 (PMC10069780; doi:10.1371/journal.pone.0283845)
Supplement: S1 Table — (DOCX) [file pone.0283845.s002.docx]

**S1 Table:** Search strategy

**SEARCH STRATEGY PUBMED – DATE 16 FEB 2022 18:31**

| **TERMS** | **SEARCH** |
| --- | --- |
| OCULAR TOXOPLASMOSIS (POPULATION)  Results: 2,650 | "toxoplasmosis, ocular"[MeSH Terms] OR "toxoplasmosis, ocular"[MeSH Terms] OR (("toxoplasm"[All Fields] OR "toxoplasma"[MeSH Terms] OR "toxoplasma"[All Fields] OR "toxoplasmal"[All Fields] OR "toxoplasmic"[All Fields] OR "toxoplasms"[All Fields]) AND "chorioretinitis"[MeSH Terms]) OR (("toxoplasm"[All Fields] OR "toxoplasma"[MeSH Terms] OR "toxoplasma"[All Fields] OR "toxoplasmal"[All Fields] OR "toxoplasmic"[All Fields] OR "toxoplasms"[All Fields]) AND "chorioretinitis"[MeSH Terms]) OR ("toxoplasmosis, ocular"[MeSH Terms] OR ("toxoplasmosis"[All Fields] AND "ocular"[All Fields]) OR "ocular toxoplasmosis"[All Fields] OR ("ocular"[All Fields] AND "toxoplasmosis"[All Fields])) OR ("toxoplasmosis, ocular"[MeSH Terms] OR ("toxoplasmosis"[All Fields] AND "ocular"[All Fields]) OR "ocular toxoplasmosis"[All Fields] OR ("toxoplasmosis"[All Fields] AND "ocular"[All Fields]) OR "toxoplasmosis ocular"[All Fields]) OR ("toxoplasmosis, ocular"[MeSH Terms] OR ("toxoplasmosis"[All Fields] AND "ocular"[All Fields]) OR "ocular toxoplasmosis"[All Fields] OR ("ocular"[All Fields] AND "toxoplasmoses"[All Fields]) OR "ocular toxoplasmoses"[All Fields]) |
| BLINDNESS (OUTCOME)  Results: 51,517 | "Blindness"[MeSH Terms] OR ("Blindness"[MeSH Terms] OR "Blindness"[All Fields] OR ("Blindness"[All Fields] AND "transient"[All Fields]) OR "blindness transient"[All Fields]) OR ("Blindness"[MeSH Terms] OR "Blindness"[All Fields] OR ("transient"[All Fields] AND "Blindness"[All Fields]) OR "transient blindness"[All Fields]) OR ("Blindness"[MeSH Terms] OR "Blindness"[All Fields] OR ("Blindness"[All Fields] AND "acquired"[All Fields]) OR "blindness acquired"[All Fields]) OR ("Blindness"[MeSH Terms] OR "Blindness"[All Fields] OR ("acquired"[All Fields] AND "Blindness"[All Fields]) OR "acquired blindness"[All Fields]) OR ("Blindness"[MeSH Terms] OR "Blindness"[All Fields] OR ("Blindness"[All Fields] AND "monocular"[All Fields]) OR "blindness monocular"[All Fields]) OR ("Blindness"[MeSH Terms] OR "Blindness"[All Fields] OR ("monocular"[All Fields] AND "Blindness"[All Fields]) OR "monocular blindness"[All Fields]) OR ("Blindness"[MeSH Terms] OR "Blindness"[All Fields] OR ("sudden"[All Fields] AND "visual"[All Fields] AND "loss"[All Fields]) OR "sudden visual loss"[All Fields]) OR ("Blindness"[MeSH Terms] OR "Blindness"[All Fields] OR ("sudden"[All Fields] AND "visual"[All Fields] AND "losses"[All Fields])) OR ("Blindness"[MeSH Terms] OR "Blindness"[All Fields] OR ("visual"[All Fields] AND "loss"[All Fields] AND "sudden"[All Fields])) OR ("Blindness"[MeSH Terms] OR "Blindness"[All Fields] OR ("Blindness"[All Fields] AND "bilateral"[All Fields]) OR "blindness bilateral"[All Fields]) OR ("Blindness"[MeSH Terms] OR "Blindness"[All Fields] OR ("Blindness"[All Fields] AND "complete"[All Fields]) OR "blindness complete"[All Fields]) OR ("Blindness"[MeSH Terms] OR "Blindness"[All Fields] OR ("complete"[All Fields] AND "Blindness"[All Fields]) OR "complete blindness"[All Fields]) OR ("Blindness"[MeSH Terms] OR "Blindness"[All Fields] OR ("bilateral"[All Fields] AND "Blindness"[All Fields]) OR "bilateral blindness"[All Fields]) OR ("Blindness"[MeSH Terms] OR "Blindness"[All Fields] OR ("bilateral"[All Fields] AND "blindnesses"[All Fields])) OR ("Blindness"[MeSH Terms] OR "Blindness"[All Fields] OR ("Blindness"[All Fields] AND "unilateral"[All Fields]) OR "blindness unilateral"[All Fields]) OR ("Blindness"[MeSH Terms] OR "Blindness"[All Fields] OR ("unilateral"[All Fields] AND "Blindness"[All Fields]) OR "unilateral blindness"[All Fields]) OR ("Blindness"[MeSH Terms] OR "Blindness"[All Fields] OR ("Blindness"[All Fields] AND "legal"[All Fields]) OR "blindness legal"[All Fields]) OR ("Blindness"[MeSH Terms] OR "Blindness"[All Fields] OR ("legal"[All Fields] AND "Blindness"[All Fields]) OR "legal blindness"[All Fields]) |
| LOW VISION (OUTCOME)  Results: 36,614 | "vision, low"[MeSH Terms] OR ("vision, low"[MeSH Terms] OR ("vision"[All Fields] AND "low"[All Fields]) OR "low vision"[All Fields] OR ("low"[All Fields] AND "vision"[All Fields])) OR ("vision, low"[MeSH Terms] OR ("vision"[All Fields] AND "low"[All Fields]) OR "low vision"[All Fields] OR ("vision"[All Fields] AND "reduced"[All Fields]) OR "vision reduced"[All Fields]) OR ("vision, low"[MeSH Terms] OR ("vision"[All Fields] AND "low"[All Fields]) OR "low vision"[All Fields] OR ("reduced"[All Fields] AND "vision"[All Fields]) OR "reduced vision"[All Fields]) OR ("vision, low"[MeSH Terms] OR ("vision"[All Fields] AND "low"[All Fields]) OR "low vision"[All Fields] OR ("vision"[All Fields] AND "subnormal"[All Fields]) OR "vision subnormal"[All Fields]) OR ("vision, low"[MeSH Terms] OR ("vision"[All Fields] AND "low"[All Fields]) OR "low vision"[All Fields] OR ("subnormal"[All Fields] AND "vision"[All Fields]) OR "subnormal vision"[All Fields]) OR ("vision, low"[MeSH Terms] OR ("vision"[All Fields] AND "low"[All Fields]) OR "low vision"[All Fields] OR ("vision"[All Fields] AND "diminished"[All Fields]) OR "vision diminished"[All Fields]) OR ("vision, low"[MeSH Terms] OR ("vision"[All Fields] AND "low"[All Fields]) OR "low vision"[All Fields] OR ("diminished"[All Fields] AND "vision"[All Fields]) OR "diminished vision"[All Fields]) |
| RECURRENCES (OUTCOME)  Results: 895,762 | "Recurrence"[MeSH Terms] OR "recurrance"[All Fields] OR "Recurrence"[MeSH Terms] OR "Recurrence"[All Fields] OR "recurrences"[All Fields] OR "recurrencies"[All Fields] OR "recurrency"[All Fields] OR "recurrent"[All Fields] OR "recurrently"[All Fields] OR "recurrents"[All Fields] OR "recrudesce"[All Fields] OR "recrudesced"[All Fields] OR "recrudescent"[All Fields] OR "recrudescing"[All Fields] OR "Recurrence"[MeSH Terms] OR "Recurrence"[All Fields] OR "recrudescence"[All Fields] OR "recrudescences"[All Fields] OR "recrudesce"[All Fields] OR "recrudesced"[All Fields] OR "recrudescent"[All Fields] OR "recrudescing"[All Fields] OR "Recurrence"[MeSH Terms] OR "Recurrence"[All Fields] OR "recrudescence"[All Fields] OR "recrudescences"[All Fields] OR "Recurrence"[MeSH Terms] OR "Recurrence"[All Fields] OR "relapse"[All Fields] OR "relapses"[All Fields] OR "relapsing"[All Fields] OR "relapsed"[All Fields] OR "relapser"[All Fields] OR "relapsers"[All Fields] OR "Recurrence"[MeSH Terms] OR "Recurrence"[All Fields] OR "relapse"[All Fields] OR "relapses"[All Fields] OR "relapsing"[All Fields] OR "relapsed"[All Fields] OR "relapser"[All Fields] OR "relapsers"[All Fields] |
| OCULAR TOXOPLASMOSIS AND (BLINDNESS OR LOW VISION OR RECURRENCES)  Results: 469 | ("toxoplasmosis, ocular"[MeSH Terms] OR "toxoplasmosis, ocular"[MeSH Terms] OR (("toxoplasm"[All Fields] OR "toxoplasma"[MeSH Terms] OR "toxoplasma"[All Fields] OR "toxoplasmal"[All Fields] OR "toxoplasmic"[All Fields] OR "toxoplasms"[All Fields]) AND "chorioretinitis"[MeSH Terms]) OR (("toxoplasm"[All Fields] OR "toxoplasma"[MeSH Terms] OR "toxoplasma"[All Fields] OR "toxoplasmal"[All Fields] OR "toxoplasmic"[All Fields] OR "toxoplasms"[All Fields]) AND "chorioretinitis"[MeSH Terms]) OR ("toxoplasmosis, ocular"[MeSH Terms] OR ("toxoplasmosis"[All Fields] AND "ocular"[All Fields]) OR "ocular toxoplasmosis"[All Fields] OR ("ocular"[All Fields] AND "toxoplasmosis"[All Fields])) OR ("toxoplasmosis, ocular"[MeSH Terms] OR ("toxoplasmosis"[All Fields] AND "ocular"[All Fields]) OR "ocular toxoplasmosis"[All Fields] OR ("toxoplasmosis"[All Fields] AND "ocular"[All Fields]) OR "toxoplasmosis ocular"[All Fields]) OR ("toxoplasmosis, ocular"[MeSH Terms] OR ("toxoplasmosis"[All Fields] AND "ocular"[All Fields]) OR "ocular toxoplasmosis"[All Fields] OR ("ocular"[All Fields] AND "toxoplasmoses"[All Fields]) OR "ocular toxoplasmoses"[All Fields])) AND ("Blindness"[MeSH Terms] OR ("Blindness"[MeSH Terms] OR "Blindness"[All Fields] OR ("Blindness"[All Fields] AND "transient"[All Fields]) OR "blindness transient"[All Fields]) OR ("Blindness"[MeSH Terms] OR "Blindness"[All Fields] OR ("transient"[All Fields] AND "Blindness"[All Fields]) OR "transient blindness"[All Fields]) OR ("Blindness"[MeSH Terms] OR "Blindness"[All Fields] OR ("Blindness"[All Fields] AND "acquired"[All Fields]) OR "blindness acquired"[All Fields]) OR ("Blindness"[MeSH Terms] OR "Blindness"[All Fields] OR ("acquired"[All Fields] AND "Blindness"[All Fields]) OR "acquired blindness"[All Fields]) OR ("Blindness"[MeSH Terms] OR "Blindness"[All Fields] OR ("Blindness"[All Fields] AND "monocular"[All Fields]) OR "blindness monocular"[All Fields]) OR ("Blindness"[MeSH Terms] OR "Blindness"[All Fields] OR ("monocular"[All Fields] AND "Blindness"[All Fields]) OR "monocular blindness"[All Fields]) OR ("Blindness"[MeSH Terms] OR "Blindness"[All Fields] OR ("sudden"[All Fields] AND "visual"[All Fields] AND "loss"[All Fields]) OR "sudden visual loss"[All Fields]) OR ("Blindness"[MeSH Terms] OR "Blindness"[All Fields] OR ("sudden"[All Fields] AND "visual"[All Fields] AND "losses"[All Fields])) OR ("Blindness"[MeSH Terms] OR "Blindness"[All Fields] OR ("visual"[All Fields] AND "loss"[All Fields] AND "sudden"[All Fields])) OR ("Blindness"[MeSH Terms] OR "Blindness"[All Fields] OR ("Blindness"[All Fields] AND "bilateral"[All Fields]) OR "blindness bilateral"[All Fields]) OR ("Blindness"[MeSH Terms] OR "Blindness"[All Fields] OR ("Blindness"[All Fields] AND "complete"[All Fields]) OR "blindness complete"[All Fields]) OR ("Blindness"[MeSH Terms] OR "Blindness"[All Fields] OR ("complete"[All Fields] AND "Blindness"[All Fields]) OR "complete blindness"[All Fields]) OR ("Blindness"[MeSH Terms] OR "Blindness"[All Fields] OR ("bilateral"[All Fields] AND "Blindness"[All Fields]) OR "bilateral blindness"[All Fields]) OR ("Blindness"[MeSH Terms] OR "Blindness"[All Fields] OR ("bilateral"[All Fields] AND "blindnesses"[All Fields])) OR ("Blindness"[MeSH Terms] OR "Blindness"[All Fields] OR ("Blindness"[All Fields] AND "unilateral"[All Fields]) OR "blindness unilateral"[All Fields]) OR ("Blindness"[MeSH Terms] OR "Blindness"[All Fields] OR ("unilateral"[All Fields] AND "Blindness"[All Fields]) OR "unilateral blindness"[All Fields]) OR ("Blindness"[MeSH Terms] OR "Blindness"[All Fields] OR ("Blindness"[All Fields] AND "legal"[All Fields]) OR "blindness legal"[All Fields]) OR ("Blindness"[MeSH Terms] OR "Blindness"[All Fields] OR ("legal"[All Fields] AND "Blindness"[All Fields]) OR "legal blindness"[All Fields]) OR ("vision, low"[MeSH Terms] OR ("vision, low"[MeSH Terms] OR ("vision"[All Fields] AND "low"[All Fields]) OR "low vision"[All Fields] OR ("low"[All Fields] AND "vision"[All Fields])) OR ("vision, low"[MeSH Terms] OR ("vision"[All Fields] AND "low"[All Fields]) OR "low vision"[All Fields] OR ("vision"[All Fields] AND "reduced"[All Fields]) OR "vision reduced"[All Fields]) OR ("vision, low"[MeSH Terms] OR ("vision"[All Fields] AND "low"[All Fields]) OR "low vision"[All Fields] OR ("reduced"[All Fields] AND "vision"[All Fields]) OR "reduced vision"[All Fields]) OR ("vision, low"[MeSH Terms] OR ("vision"[All Fields] AND "low"[All Fields]) OR "low vision"[All Fields] OR ("vision"[All Fields] AND "subnormal"[All Fields]) OR "vision subnormal"[All Fields]) OR ("vision, low"[MeSH Terms] OR ("vision"[All Fields] AND "low"[All Fields]) OR "low vision"[All Fields] OR ("subnormal"[All Fields] AND "vision"[All Fields]) OR "subnormal vision"[All Fields]) OR ("vision, low"[MeSH Terms] OR ("vision"[All Fields] AND "low"[All Fields]) OR "low vision"[All Fields] OR ("vision"[All Fields] AND "diminished"[All Fields]) OR "vision diminished"[All Fields]) OR ("vision, low"[MeSH Terms] OR ("vision"[All Fields] AND "low"[All Fields]) OR "low vision"[All Fields] OR ("diminished"[All Fields] AND "vision"[All Fields]) OR "diminished vision"[All Fields])) OR ("Recurrence"[MeSH Terms] OR "recurrance"[All Fields] OR "Recurrence"[MeSH Terms] OR "Recurrence"[All Fields] OR "recurrences"[All Fields] OR "recurrencies"[All Fields] OR "recurrency"[All Fields] OR "recurrent"[All Fields] OR "recurrently"[All Fields] OR "recurrents"[All Fields] OR "recrudesce"[All Fields] OR "recrudesced"[All Fields] OR "recrudescent"[All Fields] OR "recrudescing"[All Fields] OR "Recurrence"[MeSH Terms] OR "Recurrence"[All Fields] OR "recrudescence"[All Fields] OR "recrudescences"[All Fields] OR "recrudesce"[All Fields] OR "recrudesced"[All Fields] OR "recrudescent"[All Fields] OR "recrudescing"[All Fields] OR "Recurrence"[MeSH Terms] OR "Recurrence"[All Fields] OR "recrudescence"[All Fields] OR "recrudescences"[All Fields] OR "Recurrence"[MeSH Terms] OR "Recurrence"[All Fields] OR "relapse"[All Fields] OR "relapses"[All Fields] OR "relapsing"[All Fields] OR "relapsed"[All Fields] OR "relapser"[All Fields] OR "relapsers"[All Fields] OR "Recurrence"[MeSH Terms] OR "Recurrence"[All Fields] OR "relapse"[All Fields] OR "relapses"[All Fields] OR "relapsing"[All Fields] OR "relapsed"[All Fields] OR "relapser"[All Fields] OR "relapsers"[All Fields])) |

**SEARCH STRATEGY VHL (Virtual Health Library) – DATE 16 FEB 2022 18:31**

| **TERMS** | **SEARCH** |
| --- | --- |
| TOXOPLASMOSIS OCULAR  CEGUERA  BAJA VISIÓN  RECURRENCIA  Results: 68 | TOXOPLASMOSIS OCULAR AND (CEGUERA OR BAJA VISIÓN OR RECURRENCIA) |

**SEARCH STRATEGY EMBASE – DATE 16 FEB 2022 18:31**

| **TERMS** | **SEARCH** |
| --- | --- |
| OCULAR TOXOPLASMOSIS  RECURRENT DISEASE  BLINDNESS  VISUAL IMPAIRMENT  LOW VISION  Results: 301 | ('ocular toxoplasmosis' OR 'toxoplasmic retinochoroiditis' OR (toxoplasmic AND chorioretinitis)) AND ('recurrent disease' OR 'visual impairment' OR blindness OR 'low vision') |

**SEARCH STRATEGY OVID Cochrane Library – DATE 23 FEB 2022 13:19**

| **TERMS** | **SEARCH** |
| --- | --- |
| Ocular toxoplasmosis  toxoplasmic retinochoroiditis  toxoplasmic  chorioretinitis  Recurrence  Recurrence  Recurrences  Blindness  Low Vision  vision, low  Visual Impairments  Visual impaired  Results: 149 | (Ocular toxoplasmosis OR toxoplasmic retinochoroiditis OR (toxoplasmic AND chorioretinitis)) AND (Recurrence OR Recurrence OR Recurrences OR Blindness OR Low Vision OR vision, low OR Visual Impairments OR Visual impaired) |

**SEARCH STRATEGY Scopus – DATE 23 FEB 2022 13:19**

| **TERMS** | **SEARCH** |
| --- | --- |
| Ocular toxoplasmosis  toxoplasmic retinochoroiditis  toxoplasmic  chorioretinitis  Recurrence  Recurrence  Recurrences  Blindness  Low Vision  vision, low  Visual Impairments  Visual impaired  Results: 110 | ALL ( ( ocular AND toxoplasmosis ) AND ( recurrence OR recurrence OR recurrences OR blindness OR low AND vision OR vision, AND low OR visual AND impairments OR visual AND impaired ) ) |

**SEARCH STRATEGY DANS EASY Archive – DATE 23 FEB 2022 13:19**

| **TERMS** | **SEARCH** |
| --- | --- |
| Ocular toxoplasmosis  toxoplasmic retinochoroiditis  toxoplasmic  chorioretinitis  Recurrence  Recurrence  Recurrences  Blindness  Low Vision  vision, low  Visual Impairments  Visual impaired  Results: 21 | (Ocular toxoplasmosis OR toxoplasmic retinochoroiditis OR (toxoplasmic AND chorioretinitis)) AND (Recurrence OR Recurrence OR Recurrences OR Blindness OR Low Vision OR vision, low OR Visual Impairments OR Visual impaired) |
