# Supplementary material for: Risk factors for recurrences and visual impairment in patients with ocular toxoplasmosis: A systematic review and meta-analysis
Source: PLoS One. 2023 Apr 3;18(4):e0283845. doi: 10.1371/journal.pone.0283845 (PMC10069780; doi:10.1371/journal.pone.0283845)
Supplement: S2 Table — (DOCX) [file pone.0283845.s003.docx]

**S2 Table 1.** Risk of bias for randomized clinical trials (RCTs), the version 2 of the Cochrane risk-of-bias tool for randomized trials (RoB 2)

| **Author, year** | **Domain 1** | **Domain 2** | **Domain 3** | **Domain 4** | **Domain 5** | **Overall** |
| --- | --- | --- | --- | --- | --- | --- |
| **Fernandes Felix JP et al.  (2020 )** | Low | Low | Low | Low | Low | Low |
| **Soheilian M et al. (2011 )** | Low | Some concerns | Low | Low | Low | Low |
| **M Soheilian et al. (2005 )** | Some concerns | Low | Some concerns | Low | Some concerns | Some concerns |
| **A. Rothova et al. (1992 )** | Some concerns | Some concerns | Some concerns | Some concerns | Some concerns | Some concerns |
| **LH Bosch-Driessen et al. (2002 )** | Some concerns | Some concerns | Some concerns | High | High | Some concerns |
| **C Silveira et al. (2002 )** | Some concerns | Some concerns | Some concerns | Some concerns | Some concerns | Some concerns |
| **N Baharivan et al. (2013 )** | Low | Low | Low | Low | Low | Low |
| **LA Ghavide et al. (2017 )** | Low | Low | Low | Some concerns | Low | Low |
| **A Lashay et al  (2016 )** | Low | Low | Low | Low | Low | Low |

**S2 Table 2**. Risk of bias for Non-Randomised Studies of Interventions (ROBINS-I)

| **Author, Year** | **D1** | **D2** | **D3** | **D4** | **D5** | **D6** | **D7** | **Overall** |
| --- | --- | --- | --- | --- | --- | --- | --- | --- |
| **A Jeddi et al. (1997 )** | Serious | Serious | Serious | Serious | Serious | Serious | Serious | Serious |
| **J.C. Timsit et al. (1987 )** | Moderate | Moderate | Serious | Moderate | Moderate | Serious | Serious | Moderate |
| **E Raskin et al. (2002 )** | Low | Low | Moderate | Low | Moderate | Moderate | Moderate | Moderate |

**S2 Table 3.** Risk of Bias Instrument for Cross-Sectional ( AHRQ )

| **Author, Year** | **1) Define the source of information (survey, record review)** | **2) List inclusion and exclusion criteria for exposed and unexposed subjects (cases and controls) or refer to previous publications** | **3) Indicate time period used for identifying patients** | **4) Indicate whether or not subjects were consecutive if not population-based** | **5) Indicate if evaluators of subjective components of study were masked to other aspects of the status of the participants** | **6) Describe any assessments undertaken for quality assurance purposes (e.g., test/retest of primary outcome measurements)** | **7) Explain any patient exclusions from analysis** | **8) Describe how confounding was assessed and/or controlled.** | **9) If applicable, explain how missing data were handled in the analysis** | **10) Summarize patient response rates and completeness of data collection** | **11) Clarify what follow-up, if any, was expected and the percentage of patients for which incomplete data or follow-up was obtained** | **Overall Yes** |
| --- | --- | --- | --- | --- | --- | --- | --- | --- | --- | --- | --- | --- |
| **J Casoy et al. (2019)** | Yes | Yes | Yes | Unclear | No | Unclear | No | No | No | Yes | Yes | 5 |
| **PK Huang  et al. (2017)** | Yes | No | Yes | Unclear | No | Unclear | No | Unclear | No | Yes | No | 3 |
| **J Brydak-Godowska et al. (2015)** | Yes | No | Yes | Unclear | No | No | No | No | No | Yes | No | 3 |
| **D Kovačević-Pavićević et al. (2012)** | Yes | Unclear | Yes | Yes | No | Yes | No | Unclear | Unclear | Yes | No | 5 |
| **V Vishnevskia-Dai et al. (2019)** | Yes | Yes | Yes | Unclear | Unclear | Unclear | No | Unclear | No | Yes | Unclear | 4 |
| **A de-la-Torre et al. (2009)** | Yes | Yes | Yes | Unclear | No | Unclear | Yes | Yes | Unclear | Yes | Unclear | 6 |
| **LEH Bosch-Driessen et al. (2002)** | Yes | Yes | Yes | Yes | No | Yes | Yes | Unclear | Unclear | Yes | No | 7 |
| **GC Heringer et al. (2014)** | Yes | Yes | Yes | Yes | No | No | Yes | Yes | Unclear | Unclear | Unclear | 6 |
| **PK Borkowski et al. (2016)** | Yes | Yes | Yes | Yes | Unclear | Yes | Yes | Yes | Unclear | Yes | Unclear | 8 |
| **J G Garweg et al. (2008)** | Yes | Unclear | Yes | Yes | Unclear | Yes | Unclear | Yes | Unclear | Unclear | Unclear | 5 |
| **A de-la-Torre et al. (2008)** | Yes | Yes | Yes | Yes | Unclear | Yes | Yes | Unclear | Unclear | Unclear | Yes | 7 |
| **J Scherrer et al. (2006)** | Yes | No | Unclear | Unclear | No | No | No | Unclear | Unclear | Unclear |  | 1 |
| **M Rudzinski  et al. (2013)** | Yes | Yes | Yes | Unclear | No | Yes | Yes | No | Yes | Yes | No | 7 |
| **M. Accorinti et al. (2009)** | Yes | Unclear | Yes | Yes | No | Yes | Yes | Yes | Unclear | Yes | Yes | 8 |
| **T. Desmettre et al. (1996)** | Yes | No | Yes | No | No | No | Yes | Yes | Yes | Yes | Yes | 7 |
| **Prášil  et al. (2014)** | Yes | No | No | Yes | No | Yes | Yes | No |  | Yes | No | 5 |
| **E Majda-Stanisławska et al. (2018)** | Yes | Yes | Yes | Yes | No | Yes | No | No | No | Unclear |  | 5 |
| **A Rey et al. (2012)** | Yes | Yes | Yes | Yes | No | Yes | Yes | No | No | Yes | Yes | 8 |
| **NJS London et al. (2011)** | Yes | Yes | Yes | Yes | No | Yes | No | No | No | Yes | Yes | 7 |
| **Balıkoğlu  et al. (2009)** | Yes | Yes | Yes | Yes | No | Yes | Yes | No | Yes | Yes | Unclear | 8 |
| **M Reich et al. (2015)** | Yes | Yes | Yes | Yes | No | Yes | Yes | Yes | Yes | Yes | Yes | 10 |
| **C Silveira et al. (2011)** | Yes | No | No | Unclear | No | Yes | Unclear | No | No | Yes | No | 3 |
| **EH Bosch-Driessen  et al. (2002)** | Yes | Yes | Yes | Yes | No | No | No | No | No | Yes | Yes | 6 |
| **EH Bosch-Driessen  et al. (2000)** | Yes | Yes | Yes | Yes | No | No | Yes | No | No | Yes | No | 6 |
| **I Cochereau-Massin et al. (1992)** | Yes | Yes | Yes | No | No |  | Yes | No | Unclear | Yes | Unclear | 5 |
| **BT Naranjo Valladares et al. (2020)** | Yes | Yes | Yes | Yes | No | Unclear | Yes | No | No | Yes | Unclear | 6 |
| **FM Türkcü et al. (2016)** | Yes | Yes | Unclear | Unclear | Unclear | No | Yes | No |  | Unclear | Unclear | 3 |

**S3 Table 4.** Tool to Assess Risk of Bias in Longitudinal Symptom Research Studies Aimed at the General Population

| **Author, Year** | **1. Is the source population (sampling frame) representative of the general population?** | **2. Is the assessment of the outcome accurate both at baseline and at follow-up?** | **3. Is there little missing data?** | Definitely yes and yes |
| --- | --- | --- | --- | --- |
| **RE De Angelis et al. (2021)** | Probably no | Definitely yes (low risk of bias) | Probably yes | 2 |
| **S Velasco-Velásquez et al. (2020)** | Probably no | Definitely yes (low risk of bias) | Probably no | 1 |
| **GF Oliver et al. (2022)** | Probably yes | Probably yes | Probably yes | 3 |
| **BT Naranjo Valladares et al. (2021)** | Definitely no (high risk of bias) | Definitely yes (low risk of bias) | Probably no | 1 |

**S2 Table 5.** Tool to Assess Risk of Bias in Cohort Studies

| **Author, Year** | **1. Was selection of exposed and non-exposed cohorts drawn from the same population?** | **2. Can we be confident in the assessment of exposure?** | **3. Can we be confident that the outcome of interest was not present at start of study?** | **4. Did the study match exposed and unexposed for all variables that are associated with the outcome of interest or did the statistical analysis adjust for these prognostic variables?** | **5. Can we be confident in the assessment of the presence or absence of prognostic factors?** | **6. Can we be confident in the assessment of outcome?** | **7. Was the follow up of cohorts adequate?** | **8. Were co-interventions similar between groups?** | Definitely yes and yes |
| --- | --- | --- | --- | --- | --- | --- | --- | --- | --- |
| **S Arruda et al. (2021 )** | Definitely yes (low risk of bias) | Probably yes | Probably no | Definitely yes (low risk of bias) | Probably yes | Definitely yes (low risk of bias) | Probably yes | Probably yes | 7 |
| **C Brandão-de-Resende  et al. (2014 )** | Definitely yes (low risk of bias) | Definitely yes (low risk of bias) | Probably yes | Probably no | Probably yes | Probably yes | Definitely yes (low risk of bias) | Probably no | 6 |
| **TEF Arantes  et al. (2015 )** | Probably yes | Probably yes | Probably yes | Probably yes | Probably yes | Probably yes | Probably yes | Probably yes | 8 |
| **M Reich et al. (2015 )** | Definitely yes (low risk of bias) | Definitely yes (low risk of bias) | Probably yes | Probably yes | Probably yes | Definitely yes (low risk of bias) | Definitely yes (low risk of bias) | Definitely no (high risk of bias) | 7 |
| **ALQC Aleixo et al. (2019 )** | Definitely yes (low risk of bias) | Definitely yes (low risk of bias) | Definitely yes (low risk of bias) | Definitely yes (low risk of bias) | Probably yes | Probably yes | Probably yes | Definitely yes (low risk of bias) | 8 |
| **GN Holland et al. (2008 )** |  | Probably yes | Probably yes | Definitely no (high risk of bias) | Probably yes | Probably yes | Probably yes |  | 5 |
| **L Phan  et al. (2007 )** | Definitely yes (low risk of bias) | Definitely yes (low risk of bias) | Definitely yes (low risk of bias) | Probably yes | Definitely yes (low risk of bias) | Definitely yes (low risk of bias) | Definitely yes (low risk of bias) | Definitely yes (low risk of bias) | 8 |
| **HK Tan et al. (2007 )** |  | Probably yes | Probably yes | Probably yes | Probably yes | Probably yes | Probably yes |  | 6 |
| **Colin J and Harie JC et al. et al. (1989 )** | Definitely yes (low risk of bias) | Definitely yes (low risk of bias) | Definitely yes (low risk of bias) | Definitely no (high risk of bias) | Probably yes | Probably yes | Probably yes |  | 6 |
| **EG Lago  et al. (2021 )** | Probably yes | Probably yes | Probably yes | Probably yes | Probably yes | Probably yes | Definitely yes (low risk of bias) | Probably yes | 8 |
| **I Tugal-Tutkun et al. (2005)** | Definitely yes (low risk of bias) | Probably yes | Probably yes | Probably yes | Probably yes | Probably yes | Probably yes | Probably yes | 8 |
| **P Labalette et al. (2002 )** | Probably yes | Probably yes | Probably yes | Probably yes | Probably yes | Probably yes | Definitely yes (low risk of bias) | Definitely yes (low risk of bias) | 8 |
| **CT Friedmann et al. (1969 )** | Definitely yes (low risk of bias) | Probably yes | Probably yes | Probably yes | Probably yes | Probably yes | Definitely yes (low risk of bias) | Probably yes | 8 |
| **HF Spalter  et al. (1966 )** | Definitely no (high risk of bias) | Probably no | Probably yes | Probably no | Probably no | Probably no | Probably no | Probably no | 1 |
| **JG Garweg et al. (2005 )** | Probably yes | Probably yes | Probably yes | Probably yes | Probably yes | Probably yes | Probably yes | Probably yes | 8 |
| **MB Mets et al. (1996)** | Definitely yes (low risk of bias) | Probably yes | Probably yes | Probably yes | Probably yes | Probably yes | Probably yes | Probably yes | 8 |
| **M Wallon et al. (2004 )** | Definitely yes (low risk of bias) | Probably yes | Definitely yes (low risk of bias) | Probably no | Probably yes | Probably yes | Probably yes | Probably yes | 7 |
| **CA Cordeiro et al. (2013 )** |  | Definitely yes (low risk of bias) | Definitely yes (low risk of bias) | Definitely yes (low risk of bias) | Definitely yes (low risk of bias) | Definitely yes (low risk of bias) | Definitely yes (low risk of bias) | Definitely yes (low risk of bias) | 7 |
| **H Ocampo Rodríguez  (2015 )** | Probably yes | Definitely yes (low risk of bias) | Definitely yes (low risk of bias) | Probably no | Probably yes | Definitely yes (low risk of bias) | Probably yes | Probably yes | 7 |
| **NN Lusambo et al. (2019)** | Probably yes | Probably yes | Definitely yes (low risk of bias) | Probably yes | Probably yes | Probably yes | Definitely no (high risk of bias) | Definitely no (high risk of bias) | 6 |
| **ALQC Aleixo et al. (2016)** | **Definitely yes (low risk of bias)** | Definitely yes (low risk of bias) | Definitely yes (low risk of bias) | Definitely yes (low risk of bias) | Definitely yes (low risk of bias) | Definitely yes (low risk of bias) | Definitely yes (low risk of bias) | Definitely yes (low risk of bias) | 8 |

**S2 Table 6.** Tool to Assess Risk of Bias in Case Control Studies

| **Author, Year** | **1. Can we be confident in the assessment of exposure?** | **2. Can we be confident that cases had developed the outcome of interest and controls had not?** | **3. Were the cases (those who were exposed and developed the outcome of interest) properly selected?** | **4. Were the controls (those who were exposed and did not develop the outcome of interest) properly selected** | **5. Were cases and controls matched according to important prognostic variables or was statistical adjustment carried out for those variables?** | Definitely yes and yes |
| --- | --- | --- | --- | --- | --- | --- |
| **A de-la-Torre et al. (2014)** | Definitely yes (low risk of bias) | Probably yes | Probably yes | Probably yes | Probably no | 4 |
| **L Shobab et al. (2013)** | Definitely yes (low risk of bias) | Probably yes | Probably yes | Probably yes | Probably yes | 5 |
| **LH Bosch-Driessen et al. (2001)** | Probably yes | Probably yes | Probably yes | Probably yes | Probably yes | 5 |
| **KF de Paula Rodrigues et al. et al. (2013)** | Probably yes | Probably yes | Probably yes | Probably yes | Probably yes | 5 |
| **CA Cordeiro et al. (2015)** | Definitely yes (low risk of bias) | Definitely yes (low risk of bias) | Definitely yes (low risk of bias) | Definitely yes (low risk of bias) | Definitely yes (low risk of bias) | 5 |
| **CA Cordeiro et al. (2007)** | Definitely yes (low risk of bias) | Definitely yes (low risk of bias) | Definitely yes (low risk of bias) | Definitely yes (low risk of bias) | Definitely yes (low risk of bias) | 5 |
| **CM Ayo et al. (2016)** | Definitely yes (low risk of bias) | Definitely yes (low risk of bias) | Definitely yes (low risk of bias) | Definitely yes (low risk of bias) | Definitely yes (low risk of bias) | 5 |
| **J Isenberg et al. (2018)** | Probably yes | Probably yes | Probably yes | Probably yes | Probably yes | 5 |
