# Supplementary material for: Risk factors for recurrences and visual impairment in patients with ocular toxoplasmosis: A systematic review and meta-analysis
Source: PLoS One. 2023 Apr 3;18(4):e0283845. doi: 10.1371/journal.pone.0283845 (PMC10069780; doi:10.1371/journal.pone.0283845)
Supplement: S3 Table — (DOCX) [file pone.0283845.s004.docx]

**S3 Table 1.** Characteristics of the Metanalyzed studies

| **Author (Year)** | **Study Design** | **Study Continent** | **OT Population** | **Age a** | **Sex n (%)a** | **Reactivations by patients** | **Visual impairment (VI) (VI / Numbers of Eyes)** | **Blindness (B) (B / Numbers of Eyes)** |
| --- | --- | --- | --- | --- | --- | --- | --- | --- |
| RE De Angelis et al. (2021)[1] | Longitudinal Cross-sectional study | South America | 721 | Mean (SD)  53.18 (15.82) years | Female: 450 (62.4) Male: 271 (37.6) | Nd | 14 / 48 | 2 / 48 |
| J Casoy et al. (2019)[2] | Cross sectional study | South America | 451 | Mean (SD)  31.6 (16.5) years | Female: 220 (48.8) Male: 231 (51.2) | 296 | Nd | Nd |
| S Arruda et al. (2021)[3] | Cohort | South America | 262 | ≤17 years 11.1% 1 18-64 years 79.8% ≥65 years 9.2% | Female: 139 (53.1) Male: 123 (46.9) | 85 | 58 / 133 | 35/133 |
| C Brandão-de-Resende et al. (2014)[4] | Cohort | South America | 14 | Mean (SD) [Range]  38.6 (9.99) [15-50]years | Female: 2 (14.3) Male: 12 (85.7) | 5 | 4 / 28 | 2 /28 |
| PK Huang et al. (2017)[5] | Cross sectional study | Asia and South America | 190 | Mean [Range] 32.8 [2-73] years | Female 93 (48.9%) Male 97 (51.1%). | 83 | Nd | Nd |
| TEF Arantes et al. (2015)[6] | Cohort | South America | 139 | NR | Female 73 (52.5%) Male 66 (47.5%) | 31 | Nd | Nd |
| NN Lusambo et al. (2019)[7] | Cohort | Africa | 35 | Mean (SD) 40.9 (20) years | Female 17 (48%) Male 18 (51%) | 14 | 29 / 47 | 12 / 47 |
| Fernandes Felix JP et al. (2020)[8] | Randomized controlled trial | South America | 141 | Mean (SD) 33 (13) years | Female 75 (53.2%) Male 66 (46.8%) | Nd | Nd | Nd |
| ALQC Aleixo et al. (2019)[9] | Cohort | South America | 230 | Mean (SD) [Range] 32.8 (11.38) [14-77] years | Female 112 (48.6%) Male 118 (51.4%) | Nd | Nd | 40 / 230 |
| ALQC Aleixo et al. (2016)[10] | Cohort | South America | ** | Mean (SD) [Range] 32.84 (11.38) [14-77] years | Female 112 (48.6%) Male 118 (51.4%) | 178 | Nd | Nd |
| D Kovačević-Pavićević et al. (2012)[11] | Cross sectional study | Europe | 59 | Mean (SD) [Range] 34.9 (16.6) [13-74] years | Female 34 (57.6%) Male 25 (42.4%) | 38 | Nd | Nd |
| L Shobab et al. (2013)[12] | Case control study | Europe | 104 | Nr | Nr | 56 | Nd | Nd |
| M Soheilian et al. (2011)[13] | Randomized controlled trial | Asia | 68 | Nr | Female 34 (50%) Male 34 (50%) | Nd | Nd | Nd |
| V Vishnevskia-Dai et al. (2019)[14] | Cross sectional study | Asia | 22 | Mean (SD) [Range] 29 (18) [1 week-66] years | Female 14 (64%) Male 8 (36%) | 7 | 25 | Nd |
| M Soheilian et al. (2005)[15] | Randomized controlled trial | Asia | 59 | Nr | Female 23 (38.9%) Male 36 (61.1%) | Nd | Nd | Nd |
| A de-la-Torre et al. (2009)[16] | Cross sectional study | South America | 56 | [Range] [1-60 years] | Female 35 (62.5%) Male 21 (37.5%) | 39 | Nd | Nd |
| A. Rothova et al. (1992)[17] | Randomized controlled trial | Europe | 149 | Mean 27 years | Female 68 (45.6%) Male 81 (54.4%) | Nd | Nd | Nd |
| LEH Bosch-Driessen et al. (2002)[18] | Cross sectional study | Europe | 154 | Mean (SD) [Range] 29.5 (26) [5-89] years | Female 82 (53.24%)  Male 72 (46.74%) | 92 | Nd | 39 / 204 |
| A de-la-Torre et al. (2008)[19] | Cohort | South America | 32 | Mean [Range] 32 [13-60] years | Nr | 19 | Nd | Nd |
| J Scherrer et al. (2006)[20] | Cross sectional study | Europe | 61 | Mean (SD) [Range] 36.2 (13.8) [13-75] years | Female 40 (65.5%)  Male 21 (34.5%) | Nd | 13 / 69 | Nd |
| GF Oliver et al. (2022)[21] | Longitudinal Cross sectional study | South America | 90 | Mean (SD)  37.8 (16.3) years | Female 46 (51%)  Male 44 (49%) | 55 | 26 / 90 | 31 / 90 |
| C Silveira et al. (2002)[22] | Randomized controlled trial | South America | 124 | Nr | Female 48 (38.7%)  Male 76 (61.3%) | Nd | Nd | Nd |
| M. Accorinti et al. (2009)[23] | Cross sectional study | Europe | 88 | Mean (SD)  20.4 (14.6) years | Female 49 (55.7%)  Male 39 (44.3%) | 70 | Nd | Nd |
| N Baharivan et al. (2013)[24] | Randomized controlled trial | Asia | 66 | Nr | Female 37 (55.1%)  Male 29 (43.9%) | Nd | Nd | Nd |
| A Jeddi et al. (1997)[25] | Non Randomized controlled trial | Europe | 43 | [Range] [9-48] | Female 21 (48.8%)  Male 22 (51.2%) | Nd | Nd | Nd |
| E Majda-Stanisławska et al. (2018)[26] | Cross sectional study | Europe | 34 | Mean (SD) [Range] 7.3(15.5) [1 week-18] years | Female 23 (67,64%)  Male 11 (32,35%) | 22 | Nd | Nd |
| A Rey et al. (2012)[27] | Cross sectional study | Europe-South America | 113 | Mean (SD) 34.7 (15.8) years | Female 70 (61,9%)  Male 43 (38,05%) | 98 | Nd | 30 / 133 |
| Balıkoğlu et al. (2009)[28] | Cross sectional study | Europe-Asia | 60 | Mean (SD) [Range] 28 (12.1) [9-64] years | Female 38 (63.3%)  Male 22 (36.7%) | 13 | Nd | 8 / 60 |
| BT Naranjo Valladares et al. (2021)[29] | Longitudinal Cross sectional study | Central America | 101 | Nr | Nr | 62 | Nd | Nd |
| Colin J and Harie JC et al. et al. (1989)[30] | Randomized controlled trial | Europe | 29 | [Range] [12-57] | Female 14 (48.3%)  Male 15 (51.7%) | Nd | Nd | Nd |
| EG Lago et al. (2021)[31] | Cohort | South America | 55 | Nr | Nr | 39 | Nd | Nd |
| I Tugal-Tutkun et al. (2005)[32] | Cohort | Europe | 109 | Mean (SD) [Range] 25.7 (6.8) [13-44] years | Female 52 (47.7%%)  Male 54 (52.3%) | 26 | Nd | Nd |
| P Labalette et al. (2002)[33] | Cohort | Europe | 27 | Mean (SD) [Range] 65.1 (9) [50-87] years | Female 16 (59%%)  Male 11 (41%) | 8 | Nd | Nd |
| CT Friedmann et al. (1969)[34] | Cohort | North America | 63 | Mean [Range] 25 [7-57] years | Female 33 (52.4%) Male 30 (47.6%) | 39 | Nd | Nd |
| MB Mets et al. (1996)[35] | Cohort | North America | 94 | [Range] [Birth-72 months] | Female 41 (43.6%) Male 53 (56.4%) | 19 | Nd | Nd |
| M Wallon et al. (2004)[36] | Cohort | Europe | 79 | Birth | Nr | 23 | Nd | Nd |
| BT Naranjo Valladares et al. (2020)[37] | Cross sectional study | Central America | 39 | Mean  12.8 | Female 18 (46.2%) Male 21 (53.8%) | 15 | Nd | 39 / 20 |
| H Ocampo Rodríguez (2015)[38] | Cohort | South America | 22 | Nr | Female 6 (27%) Male 16 (73%) | Nd | Nd | 16 / 22 |
| FM Türkcü et al. (2016)[39] | Cross sectional study | Europe | 17 | Mean (SD) 29.08 (5.71) years | Female 100% | Nd | 7 / 17 | 2 / 17 |

****Same population as Alexio 2019** [9]

**References:**

1. De Angelis RE, Veronese Rodrigues M de L, Passos ADC, Bollela VR, Freitas E Silva MS, Vieira BR, et al. Frequency and visual outcomes of ocular toxoplasmosis in an adult Brazilian population. Sci Rep. 2021;11: 3420. doi:10.1038/s41598-021-83051-0

2. Casoy J, Nascimento H, Silva LMP, Fernández-Zamora Y, Muccioli C, Dias JR de O, et al. Effectiveness of Treatments for Ocular Toxoplasmosis. Ocul Immunol Inflamm. 2020;28: 249–255. doi:10.1080/09273948.2019.1569242

3. Arruda S, Vieira BR, Garcia DM, Araújo M, Simões M, Moreto R, et al. Clinical manifestations and visual outcomes associated with ocular toxoplasmosis in a Brazilian population. Sci Rep. 2021;11: 3137. doi:10.1038/s41598-021-82830-z

4. Brandão-de-Resende C, Santos HH, Rojas Lagos AA, Lara CM, Arruda JSD, Marino APMP, et al. Clinical and Multimodal Imaging Findings and Risk Factors for Ocular Involvement in a Presumed Waterborne Toxoplasmosis Outbreak, Brazil(1). Emerg Infect Dis. 2020;26: 2922–2932. doi:10.3201/eid2612.200227

5. Huang PK, Jianping C, Vasconcelos-Santos DV, Arruda JSD, Dutta Majumder P, Anthony E, et al. Ocular Toxoplasmosis in Tropical Areas: Analysis and Outcome of 190 Patients from a Multicenter Collaborative Study. Ocul Immunol Inflamm. 2018;26: 1289–1296. doi:10.1080/09273948.2017.1367407

6. Arantes TEF, Silveira C, Holland GN, Muccioli C, Yu F, Jones JL, et al. Ocular Involvement Following Postnatally Acquired Toxoplasma gondii Infection in Southern Brazil: A 28-Year Experience. Am J Ophthalmol. 2015;159: 1002-1012.e2. doi:10.1016/j.ajo.2015.02.015

7. Nsiangani Lusambo N, Kaimbo Wa Kaimbo D. Profil clinique et épidémiologique de la toxoplasmose oculaire à Kinshasa. Journal Français d’Ophtalmologie. 2019;42: 900–906. doi:10.1016/j.jfo.2019.05.017

8. Fernandes Felix JP, Cavalcanti Lira RP, Grupenmacher AT, Assis Filho HLG de, Cosimo AB, Nascimento MA, et al. Long-term Results of Trimethoprim-Sulfamethoxazole Versus Placebo to Reduce the Risk of Recurrent Toxoplasma gondii Retinochoroiditis. Am J Ophthalmol. 2020;213: 195–202. doi:10.1016/j.ajo.2019.12.025

9. Aleixo ALQ do C, Vasconcelos C de Oliveira R, Cavalcanti Albuquerque M, Biancardi AL, Land Curi AL, Israel Benchimol E, et al. Toxoplasmic retinochoroiditis: The influence of age, number of retinochoroidal lesions and genetic polymorphism for IFN-γ +874 T/A as risk factors for recurrence in a survival analysis. PLoS One. 2019;14: e0211627. doi:10.1371/journal.pone.0211627

10. Aleixo ALQ do C, Curi ALL, Benchimol EI, Amendoeira MRR. Toxoplasmic Retinochoroiditis: Clinical Characteristics and Visual Outcome in a Prospective Study. PLoS Negl Trop Dis. 2016;10: e0004685. doi:10.1371/journal.pntd.0004685

11. Kovačević-Pavićević D, Radosavljević A, Ilić A, Kovačević I, Djurković-Djaković O. Clinical pattern of ocular toxoplasmosis treated in a referral centre in Serbia. Eye (Lond). 2012;26: 723–728. doi:10.1038/eye.2012.20

12. Shobab L, Pleyer U, Johnsen J, Metzner S, James ER, Torun N, et al. Toxoplasma serotype is associated with development of ocular toxoplasmosis. J Infect Dis. 2013;208: 1520–1528. doi:10.1093/infdis/jit313

13. Soheilian M, Ramezani A, Azimzadeh A, Sadoughi MM, Dehghan MH, Shahghadami R, et al. Randomized trial of intravitreal clindamycin and dexamethasone versus pyrimethamine, sulfadiazine, and prednisolone in treatment of ocular toxoplasmosis. Ophthalmology. 2011;118: 134–141. doi:10.1016/j.ophtha.2010.04.020

14. Vishnevskia-Dai V, Achiron A, Buhbut O, Berar OV, Musika AA, Elyashiv SM, et al. Chorio-retinal toxoplasmosis: treatment outcomes, lesion evolution and long-term follow-up in a single tertiary center. Int Ophthalmol. 2020;40: 811–821. doi:10.1007/s10792-019-01242-1

15. Soheilian M, Sadoughi M-M, Ghajarnia M, Dehghan MH, Yazdani S, Behboudi H, et al. Prospective randomized trial of trimethoprim/sulfamethoxazole versus pyrimethamine and sulfadiazine in the treatment of ocular toxoplasmosis. Ophthalmology. 2005;112: 1876–1882. doi:10.1016/j.ophtha.2005.05.025

16. de-la-Torre A, Rios-Cadavid AC, Cardozo-García CM, Gomez-Marín JE. Frequency and factors associated with recurrences of ocular toxoplasmosis in a referral centre in Colombia. Br J Ophthalmol. 2009;93: 1001–1004. doi:10.1136/bjo.2008.155861

17. Rothova A, Meenken C, Buitenhuis HJ, Brinkman CJ, Baarsma GS, Boen-Tan TN, et al. Therapy for ocular toxoplasmosis. Am J Ophthalmol. 1993;115: 517–523. doi:10.1016/s0002-9394(14)74456-3

18. Bosch-Driessen LEH, Berendschot TTJM, Ongkosuwito JV, Rothova A. Ocular toxoplasmosis: clinical features and prognosis of 154 patients. Ophthalmology. 2002;109: 869–878. doi:10.1016/s0161-6420(02)00990-9

19. de-la-Torre A, López-Castillo CA, Gómez-Marín JE. Incidence and clinical characteristics in a Colombian cohort of ocular toxoplasmosis. Eye (Lond). 2009;23: 1090–1093. doi:10.1038/eye.2008.219

20. Scherrer J, Iliev ME, Halberstadt M, Kodjikian L, Garweg JG. Visual function in human ocular toxoplasmosis. Br J Ophthalmol. 2007;91: 233–236. doi:10.1136/bjo.2006.100925

21. Oliver GF, Ferreira LB, Vieira BR, Arruda S, Araújo M, Carr JM, et al. Posterior segment findings by spectral-domain optical coherence tomography and clinical associations in active toxoplasmic retinochoroiditis. Sci Rep. 2022;12: 1156. doi:10.1038/s41598-022-05070-9

22. Silveira C, Belfort RJ, Muccioli C, Holland GN, Victora CG, Horta BL, et al. The effect of long-term intermittent trimethoprim/sulfamethoxazole treatment on recurrences of toxoplasmic retinochoroiditis. Am J Ophthalmol. 2002;134: 41–46. doi:10.1016/s0002-9394(02)01527-1

23. Accorinti M, Bruscolini A, Pirraglia MP, Liverani M, Caggiano C. Toxoplasmic retinochoroiditis in an Italian referral center. Eur J Ophthalmol. 2009;19: 824–830. doi:10.1177/112067210901900522

24. Baharivand N, Mahdavifard A, Fouladi RF. Intravitreal clindamycin plus dexamethasone versus classic oral therapy in toxoplasmic retinochoroiditis: a prospective randomized clinical trial. Int Ophthalmol. 2013;33: 39–46. doi:10.1007/s10792-012-9634-1

25. Jeddi A, Azaiez A, Bouguila H, Kaoueche M, Malouche S, Daghfous F, et al. [Value of clindamycin in the treatment of ocular toxoplasmosis]. J Fr Ophtalmol. 1997;20: 418–422.

26. Majda-Stanisławska E, Socha K, Sicinska J, Kuc A, Niwald A, Moll A, et al. Long-term observation of ocular toxoplasmosis in immunocompetent children. Pediatr Pol. 2018;93: 17–22. doi:10.5114/POLP.2018.74769

27. Rey A, Llorenç V, Pelegrín L, Mesquida M, Molins B, Rios J, et al. Clinical pattern of toxoplasmic retinochoroiditis in a Spanish referral center. Ophthalmologica. 2013;229: 173–178. doi:10.1159/000348740

28. Çakar Özdal MP, Balikoğlu Yilmaz M, Özdemi̇r P, Kavuncu S, Öztürk F. Aktif oküler toksoplazmoziste nüks ve sonuç görme keskinliğini etkileyen prognostik faktörler. Retina-Vitreus. 2009;17: 255–262. Available: http://search/yayin/detay/99263

29. Naranjo Valladares BT, León Sánchez MA, López MR. Retinocoroiditis toxoplásmica y la evolución del resultado visual en pacientes inmunocompetentes. Rev cuba oftalmol. 2021;34: e983–e983. Available: http://scielo.sld.cu/scielo.php?script=sci_arttext&pid=S0864-21762021000300005

30. Colin J, Harie JC. [Presumed toxoplasmic chorioretinitis: comparative study of treatment with pyrimethamine and sulfadiazine or clindamycin]. J Fr Ophtalmol. 1989;12: 161–165.

31. Lago EG, Endres MM, Scheeren MF da C, Fiori HH. Ocular Outcome of Brazilian Patients With Congenital Toxoplasmosis. Pediatr Infect Dis J. 2021;40: e21–e27. doi:10.1097/INF.0000000000002931

32. Tugal-Tutkun I, Corum I, Otük B, Urgancioglu M. Active ocular toxoplasmosis in Turkish patients: a report on 109 cases. Int Ophthalmol. 2005;26: 221–228. doi:10.1007/s10792-007-9047-8

33. Labalette P, Delhaes L, Margaron F, Fortier B, Rouland J-F. Ocular toxoplasmosis after the fifth decade. Am J Ophthalmol. 2002;133: 506–515. doi:10.1016/s0002-9394(02)01324-7

34. Friedmann CT, Knox DL. Variations in recurrent active toxoplasmic retinochoroiditis. Arch Ophthalmol. 1969;81: 481–493. doi:10.1001/archopht.1969.00990010483005

35. Mets MB, Holfels E, Boyer KM, Swisher CN, Roizen N, Stein L, et al. Eye manifestations of congenital toxoplasmosis. Am J Ophthalmol. 1997;123: 1–16. doi:10.1016/s0002-9394(14)70986-9

36. Wallon M, Kodjikian L, Binquet C, Garweg J, Fleury J, Quantin C, et al. Long-term ocular prognosis in 327 children with congenital toxoplasmosis. Pediatrics. 2004;113: 1567–1572. doi:10.1542/peds.113.6.1567

37. Naranjo Valladares BT, León Sánchez MA, Iglesias Rojas MB, Sainz Padrón L. Toxoplasmosis ocular: aspectos clínico-epidemiológicos en edad pediátrica. Rev cienc med Pinar Rio. 2020;24: e4457–e4457. Available: http://scielo.sld.cu/scielo.php?script=sci_arttext&pid=S1561-31942020000400013

38. Ocampo Domínguez HH. Manejo de toxoplasmosis ocular severa con clindamicina y triamcinolona intravitreas: reporte de 22 casos. Rev Soc Colomb Oftalmol. 2015;48: 312–321. Available: https://scopublicaciones.socoftal.com/index.php/SCO/article/view/109/111

39. Türkcü FM, Şahin A, Yüksel H, Çınar Y, Cingü K, Altındağ S, et al. Activation of toxoplasma retinochoroiditis during pregnancy and evaluation of ocular findings in newborns. International Ophthalmology. 2017;37: 559–563. doi:10.1007/s10792-016-0311-7
