# Supplementary material for: Risk factors for recurrences and visual impairment in patients with ocular toxoplasmosis: A systematic review and meta-analysis
Source: PLoS One. 2023 Apr 3;18(4):e0283845. doi: 10.1371/journal.pone.0283845 (PMC10069780; doi:10.1371/journal.pone.0283845)
Supplement: S1 Fig — Fail-Safe N analysis (Fail-safe N = 30630; P<0.001), Rank correlation test (Tau = -0.066; P = 0.650), Asymmetry (Z = -0.375; P = 0.708). Fail-Safe N analysis (Fail-safe N = 857; P <0.001), Rank correlation test (Tau = 0.333; P = 0.260), Asymmetry (Z = 1.201; P = 0.230). Fail-Safe N analysis (Fail-safe N = 1086; P <0.001), Rank correlation test (Tau = 0.212; P = 0.381), Asymmetry (Z = 2.225; P = 0.026). (DOCX) [file pone.0283845.s005.docx]

**S1 Fig 1** Frequency of recurrence segmented by continent Funnel-Plot.


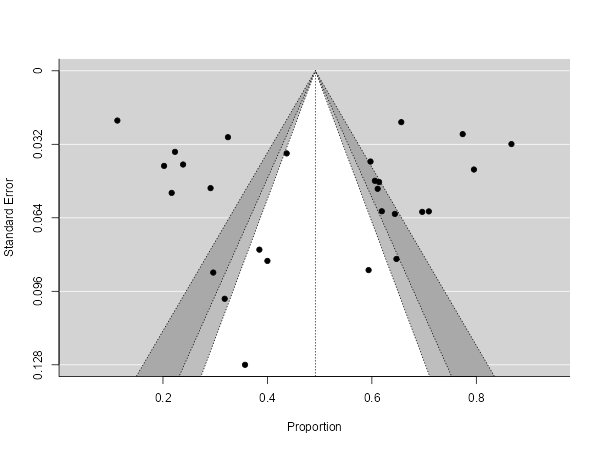


Fail-Safe N analysis (Fail-safe N=30630; *P*<0.001), Rank correlation test (Tau= -0.066 ; *P* =0.650), Asymmetry (Z=-0.375; *P*=0.708).

**S1 Fig 2** Prevalence of visual impairment OT eyes Funnel Plot


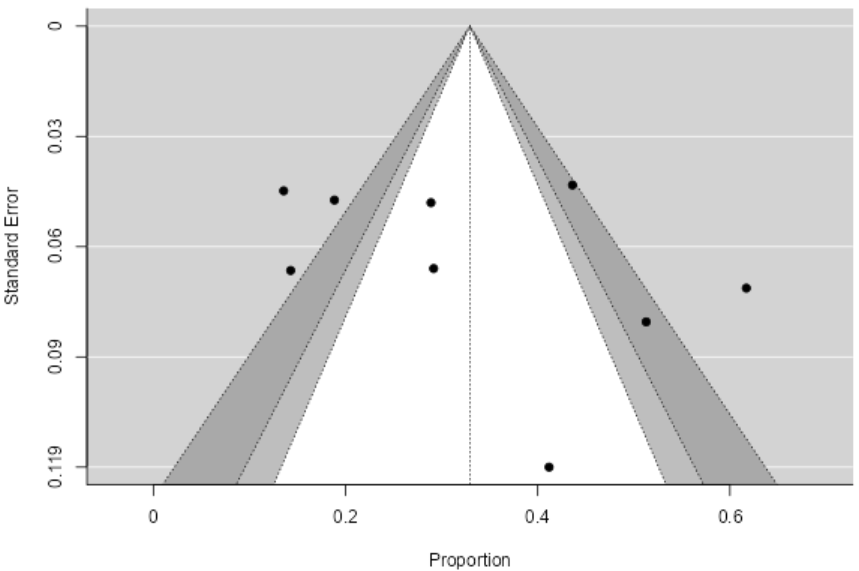


Fail-Safe N analysis (Fail-safe N=857; *P* <0.001), Rank correlation test (Tau= 0.333; *P* =0.260), Asymmetry (Z= 1.201; *P*=0.230).

**S1 Fig 3** Prevalence of blindness in OT eyes Funnel Plot
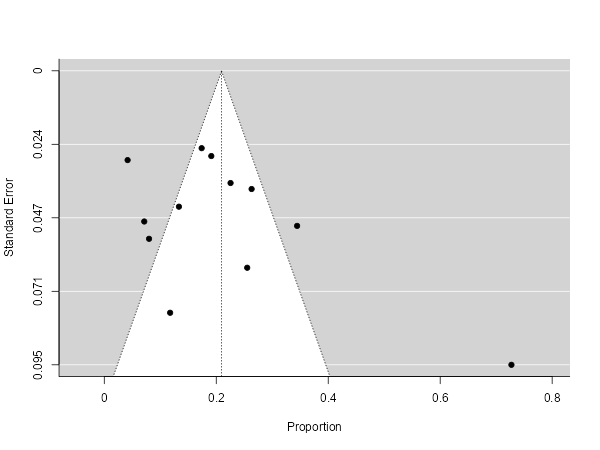


Fail-Safe N analysis (Fail-safe N=1086; *P* <0.001), Rank correlation test (Tau= 0. 212; *P* =0.381), Asymmetry (Z= 2.225; *P*=0.026).
